# Transcriptional control of central carbon metabolic flux in Bifidobacteria by two functionally similar, yet distinct LacI-type regulators

**DOI:** 10.1038/s41598-019-54229-4

**Published:** 2019-11-28

**Authors:** Noreen Lanigan, Emer Kelly, Aleksandr A. Arzamasov, Catherine Stanton, Dmitry A. Rodionov, Douwe van Sinderen

**Affiliations:** 1 School of Microbiology & APC Microbiome Ireland, University College Cork, Ireland University College Cork, Cork, Ireland; 2 Sanford Burnham Prebys Medical Discovery Institute, La Jolla, United States; 3 A.A. Kharkevich Institute for Information Transmission Problems, Russian Academy of Sciences, Moscow, Russia; 4 Teagasc Food Research Centre, Moorepark, Fermoy, Cork, Ireland

**Keywords:** Bacterial genomics, Cellular microbiology, Bacterial genetics

## Abstract

Bifidobacteria resident in the gastrointestinal tract (GIT) are subject to constantly changing environmental conditions, which require rapid adjustments in gene expression. Here, we show that two predicted LacI-type transcription factors (TFs), designated AraQ and MalR1, are involved in regulating the central, carbohydrate-associated metabolic pathway (the so-called phosphoketolase pathway or bifid shunt) of the gut commensal *Bifidobacterium breve* UCC2003. These TFs appear to not only control transcription of genes involved in the bifid shunt and each other, but also seem to commonly and directly affect transcription of other TF-encoding genes, as well as genes related to uptake and metabolism of various carbohydrates. This complex and interactive network of AraQ/MalR1-mediated gene regulation provides previously unknown insights into the governance of carbon metabolism in bifidobacteria.

## Introduction

The microbial communities within the human gastrointestinal tract (GIT), commonly referred to as the gut microbiota, have enjoyed intense scientific scrutiny and are predominantly made up of members of the bacterial phyla Bacteroidetes, Firmicutes, Proteobacteria and Actinobacteria^1,2^. Of particular interest is the genus *Bifidobacterium*, a member of the Actinobacteria phylum, that represent Gram-positive, non-motile, non-spore forming, saccharoclastic anaerobic bacteria that commonly colonize the gut of a wide variety of animals, including birds, insects and mammals^3^. Several bifidobacterial strains/species are designated as probiotic, which means that when such bacteria are administered in adequate and viable numbers, they confer a health benefit to their (human) host^4^. These benefits include, among others, modulation of the host immune response^5^, mitigation of lactose intolerance, the ability to lower serum cholesterol levels in humans^6–8^, pathogen inhibition/exclusion^9^ and prophylaxis of tumour growth in certain cancers^10–12^. Bifidobacteria are among the first and predominant colonisers of the infant GIT, and persist throughout adulthood though at lower relative abundance (compared to the infant gut), with a further reduction in the elderly gut^13–15^.

Approximately 8–15% of the bifidobacterial coding capacity is dedicated to carbohydrate uptake and metabolism, depending on species and functional gene assignment^16,17^. These estimates are consistent with the well-documented ability of bifidobacteria to utilise a diverse array of carbohydrates^16,18^. This remarkable genetic dedication to a particular metabolic behaviour probably reflects the importance of carbohydrate utilisation for bifidobacterial gut colonization and persistence. Just as important is their ability to control expression of the genes involved in the associated metabolic pathways in order to prevent wasting energy and resources on something which is unnecessary or inefficient. This is a widespread phenomenon, exemplified by the *lac* operon of *Escherichia coli*, which is only switched on if the substrate lactose is present and if a better (i.e. more energy generating) substrate such as glucose is absent^19^.

Additionally, carbon flux control enables bifidobacteria to balance energy and biosynthetic demands, thereby allowing optimal growth and redox balance management, as based on environmental conditions. This control is of crucial importance to bifidobacteria, as carbohydrate metabolism is their sole mechanism to produce ATP by substrate phosphorylation^20^, while metabolic intermediates of carbon metabolism (such as pyruvate) are used as precursors for a range of biosynthetic processes. Carbohydrate metabolism of bifidobacteria is performed by a rather unique pathway, the so-called phosphoketolase pathway or bifid shunt, which allows this taxonomic group to produce more energy in the form of ATP from carbohydrates than fermentative pathways employed by other bacteria^21^. From 1 mol of glucose the bifid shunt theoretically yields 2.5 mol ATP, along with 1.5 mol of acetate and 1 mol of lactate^22^, whereas for example homofermentative lactic acid bacteria (LAB) produce 2 mol of ATP and 2 mol of lactic acid per mol of glucose^23^. This superior ATP production ability is due to the presence of a phosphoketolase called X5P/F6P phosphoketolase (XFPK, encoded by *xfpK*) which is unique in exhibiting comparable affinities for both xylulose 5-P (X5P) and fructose 6-P (F6P)^24^. The bifid shunt has been investigated in terms of its enzymatic functions in animal-derived species, such as *Bifidobacterium globosum*^25,26^, and it has been established that the bifid shunt is indeed the sole carbohydrate metabolism pathway to satisfy all energy demands in *Bifidobacterium animalis* subsp. *lactis* Bb-12 when grown on either glucose or lactose^24^. All members of the *Bifidobacteriaceae* family are said to possess *xfpK* homologs, and thus employ the bifid shunt^27^. Phylogenetic and protein modelling studies have recently suggested that the bifidobacterial XFPK enzyme is related to that found in members of the Coriobacteriales order and that *xfpK* was horizontally transferred between (an ancestor of) these two groups^21,28,29^. Another interesting difference between bifidobacteria (and now possibly Coriobacteriales) and other bacteria is that they lack a number of enzymes, such as phosphofructokinase, which are crucial to control central carbohydrate metabolism^30^.

**Figure 1:**
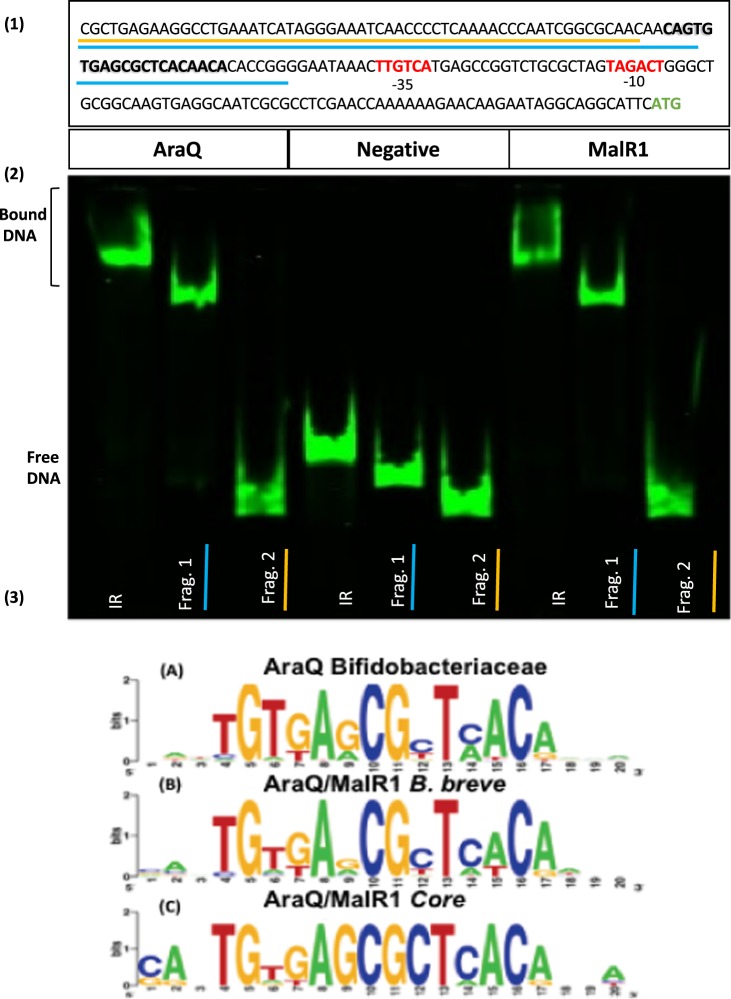
Fragmentation analysis and AraQ/MalR1 binding. Panel (1) depicts the promoter region of *pyk* (Bbr_0757) used for fragmentation analysis. −35 and −10 sites are indicated in red and the ATG start codon is indicated in green. The TFBS is indicated in bold. The fragment including the TFBS (Frag. 1) is underlined in blue, the fragment excluding the TFBS (Frag. 2) is underlined in yellow, while the full fragment is referred to as IR. Panel (2) EMSA to investigate AraQ and MalR1 abilities to bind to Bbr_0757 promoter region fragments (IR, Frag 1 and Frag 2). All reactions contain 0.5 nM Ird labelled DNA and 150 nM of either AraQ or MalR1 protein, while negative reactions contain 0 nM protein. Uncropped gel image available in Supplemental Fig. S5. Panel (3), (**A**) Illustrates consensus AraQ/MalR1 binding motif across the *Bifidobacteriaceae* family (**B**) Depicts the binding motif of AraQ/MalR1 in *B*. *breve*. (**C**) Depicts the AraQ/MalR1 binding motif for core members of the regulon.

Various carbohydrate utilisation pathways in bifidobacteria have been characterised^31–39^. Several carbohydrate metabolism-related LacI-type regulators are known in *Bifidobacterium breve* UCC2003^34–37^ and are typically involved in local (i.e. the gene encoding the LacI-type regulator is located in the vicinity of the genes it regulates) transcriptional regulation of genes that are responsible for the metabolism of a particular carbohydrate. These LacI-type transcription factors (TFs) typically limit (and are then called repressors), yet may sometimes enhance (then designated as activators), access or progression of the RNA polymerase to the promoter region of the regulated gene. This ability to act as a repressor or activator depends on a number of factors such as the location of the Transcription Factor Binding Site (TFBs) of the TF with respect to the -10/-35 sequences of the promoter region and the presence or absence of effector molecules, which may bind to the TF to alter its conformation and consequently its DNA-binding ability (See Fig. 1 Panel 3 for an example of a LacI TFBS). The binding state of the TF must be in accordance with the external and/or cytoplasmic environment, and, as mentioned, bacteria achieve this through effector molecules. In the case of carbohydrate utilisation such effector molecules may be a carbohydrate or metabolic intermediate.

It had previously been predicted that the *B*. *breve* UCC2003-encoded LacI-type regulators (termed MalR1, MalR2, MalR3, MalR5 and MalR6) are involved in the metabolism of starch-like carbohydrates, while AraQ was envisaged to be responsible for transcriptional control of genes encoding a number of bifid shunt enzymes^40^. We show here that two of the previously identified LacI-type TFs appear to be directly involved in transcriptionally regulating genetic components of the bifid shunt, as well as genes involved in a range of specific carbohydrate metabolic pathways, thereby revealing a previously undiscovered global carbohydrate control network, which is predicted to operate in many bifidobacterial species.

## Results

### Identification and genetic analysis of *araQ* and *malR1* on the *B. breve* UCC2003 genome

This study was initially aimed at the functional characterization of six previously described LacI-type transcription factors (TFs), namely AraQ, MalR1, MalR2, MalR3, MalR5 and MalR6 along with their proposed regulation of central metabolic pathways and malto-oligosaccharide utilisation pathways in *B*. *breve* UCC2003^40^. In the course of our experiments we found that MalR1 was in fact functionally more similar to AraQ and for this reason our experimental scope was narrowed to specifically characterise AraQ and MalR1. The encoded products of *araQ* and *malR1* represent proteins of 371 and 338 amino acids, respectively, which exhibit 26% overall identity to each other (91% query coverage). Their respective DNA binding domains (encompassing residues 11–57 for AraQ and residues 3–47 for MalR1) exhibit a higher level of identity at 49% (93% query coverage) (BlastP). This may be a reflection of the conserved nature of the DNA-binding helix-turn-helix regions of LacI-type proteins in general^41^, coupled to the finding that, as will be outlined below, AraQ and MalR1 recognize (near) identical operator sequences.

### *In vitro* characterisation of the regulons of AraQ & MalR1 by EMSA analysis

**Figure 2:**
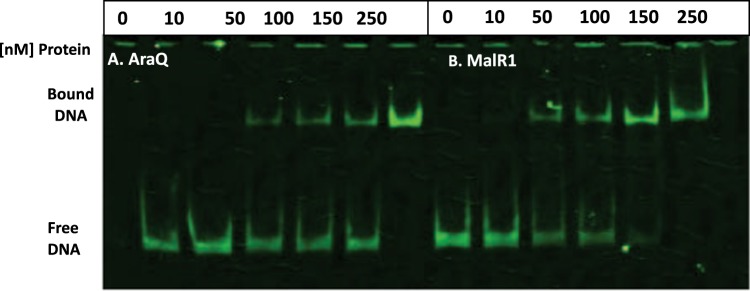
EMSA analysis with AraQ/MalR1. EMSA analysis carried out with increasing concentrations (0, 10, 50, 100, 150, 250 nM) of purified AraQ (panel A) or MalR1 (panel B) incubated with 0.5 nM Ird-labelled DNA fragment encompassing the Bbr_1233 (*gap*) promoter region. Uncropped gel image available in Supplemental Fig. S4.

**Figure 3:**
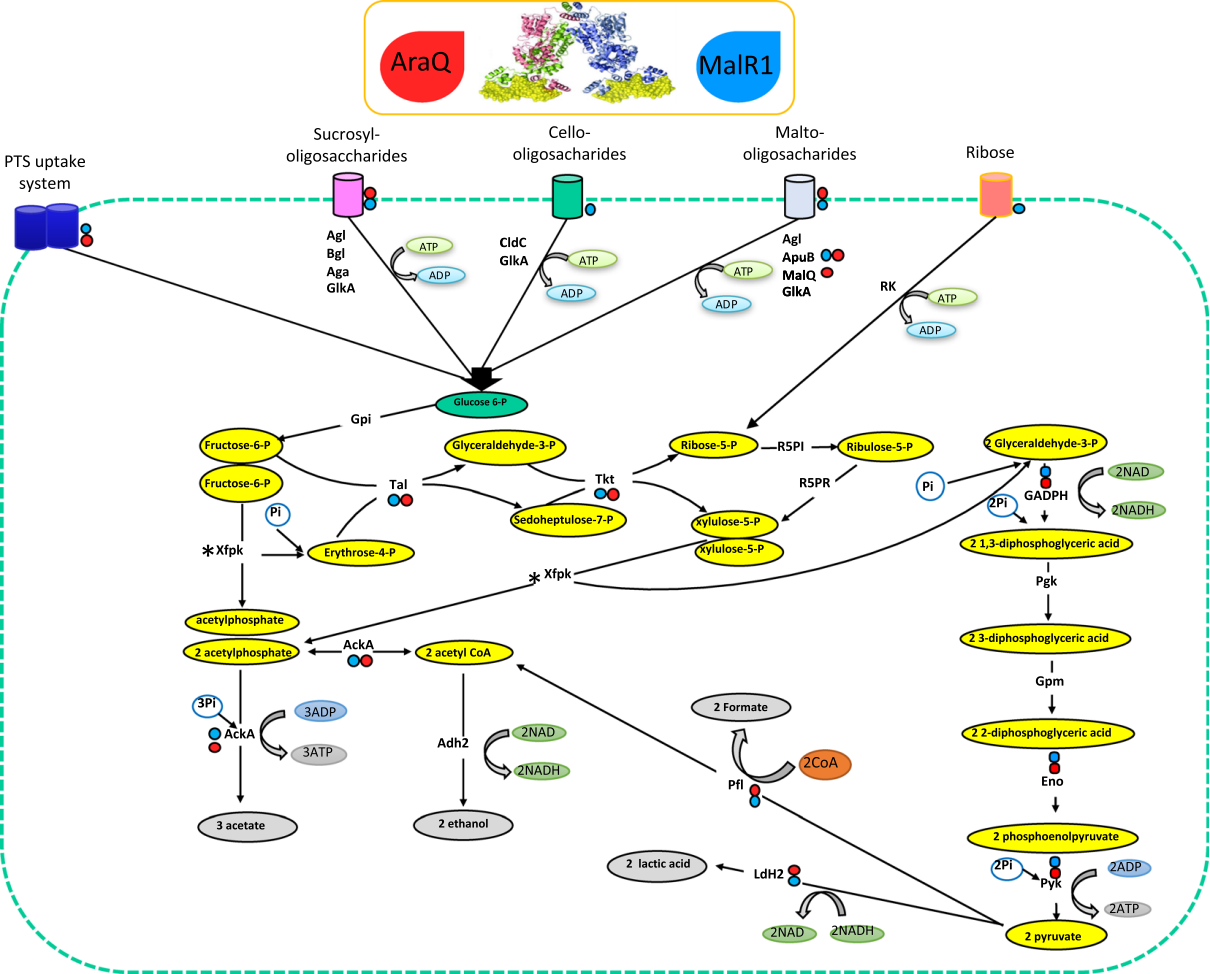
Schematic of AraQ and MalR1 effects on Central metabolism. Schematic of predicted central metabolism steps for carbohydrate metabolism in *B*. *breve* UCC2003. The XFPK enzymatic steps are indicated by an asterisk. As determined by EMSA analysis in Table 1, enzymatic steps which are under the regulation of AraQ and MalR1 are indicated by a red or blue dot, respectively. Abbreviation: Agl, alpha glucosidase; Bgl, beta gluosidase; GlkA, glucokinase; CldC, beta glucosidase; ApuB, amylopullunase; MalQ, glucanotransferase; Rk, ribokinase; Gpi, glucose- 6-p isomerase; Tal, transaldolase; Tkt, transketolase; R5PI, ribose-5-P isomerase; R5PR, ribose-5-P reductase; GADPH, glyceraldehyde-3-phosphate dehydrogenase; Xfpk, Xylose-5-P/Fructose-6-P phosphoketolase; AckA, acetate kinase; Ald2, alcohol dehydrogenase 2; Pgk, phosphoglycerate kinase; Gpm, phophoglycerate mutase; Eno, enolase; Pyk, pyruvate kinase; Pfl, pyruvate formate lyase; ldh2, lactate dehydrogenase 2.

In order to assess AraQ and MalR1 interaction with their possible operator sequences, electrophoretic mobility shift assays (EMSAs) were carried out using DNA fragments encompassing upstream regions of genes corresponding to the previously predicted AraQ and MalR1 regulons^40^, and to a number of (differentially transcribed) genes that were identified in microarray analyses involving insertion mutants of *araQ* and *malR1* (discussed below). Obtaining purified and active AraQ or MalR1 protein from *B*. *breve* initially proved to be troublesome, in line with similar issues observed for other bifidobacterial LacI-type proteins^34–37,42^. We adapted our original protocol so as to allow purification of active (i.e. DNA-binding competent) AraQ and MalR1 (see Materials and Methods), which were tested for their ability to bind to DNA sequences encompassing the operator sequence or transcription factor binding site (TFBS) previously predicted for AraQ^40^. The ability of AraQ and MalR1 to bind DNA fragments was graded utilising a non-linear, arbitrary scale (see Materials and Methods). This EMSA analysis revealed that AraQ and MalR1 bind with varying degrees of affinity to the promoter regions of 23 and 20 genes, respectively, out of a total of 33 DNA regions tested. Figure 2 presents an example of complete DNA fragment binding, while additional examples can be found in Supplemental Fig. S1. Table 1 summarises all obtained EMSA results, while Fig. 3 is a schematic representation of the results obtained in this study. Interestingly, some of the promoter regions to which AraQ/MalR1 did not appear to bind, are associated with genes that display differential transcription in the microarray analysis of the corresponding *araQ*/*malR1* mutants (see below), indicating that such differential transcription reflects indirect regulatory effects.

**Table 1:** EMSA Analysis of recombinant AraQ and MalR1 proteins.

Gene	Locus tag; Bbr	Function (predicted or experimentally validated)	AraQ	MalR1	High level of transcription on glucose (H) and essential (E)	Repression (R) or Activation (A) by AraQ & MalR1
**Predicted Maltose/Maltodextrin Utilization Regulon**						
*malG2 (*F2*)	0027	Sugar ABC transporter, permease	++	−	−	R
*malR5	0032	Transcriptional regulator, LacI family	+	+	−	−
*malE2	0033	Sugar ABC transporter, solute binding protein	+	+	−	−
*glgP1*	0060	Glycogen phosphorylase	++	−	H	R
*agl3*	0111	α-glucosidase	−	−	−	−
*malR6	0112	Transcriptional regulator, LacI family	−	++	−	−
Bbr_0113	0113	Sortase	++	++	−	−
*malQ*	0116	Glucanotransferase	+	−	−	R
*agl4/ malE1	0117/0118	α-glucosidase/Sugar ABC transporter, solute binding protein	++	++	−	R
*malR3	0122	Transcriptional regulator, LacI family	+	+	−	−
*apuB	0123	Amylopullulanase	+	+	−	−
*malF / malR1	1845/1846	Sugar ABC transporter, permease/Transcriptional regulator, LacI family	++	++	−	R
*malE*	1847	Sugar ABC transporter, solute binding protein	++	−	−	A
**Predicted Central Metabolic Regulon**						
*araQ*	0411	Transcriptional regulator, LacI family	+++	+++	−	R
*eno*	0725	Enolase	+++	+++	H, E	A
*carD*	0747	CarD-like transcriptional regulator	+++	++	H, E	A
*pyk*	0757	Pyruvate kinase	+++	+++	H	A
*pflB - pflA*	0787	Formate acetyl transferase - Pyruvate formate-lyase activating enzyme	++	+++	H	A
*tkt - tal*	1002	Transketolase - Transaldolase	++	++	H, E	A
*gap*	1233	Glyceraldehyde phosphatase	+++	+++	H, E	A
*ldh2*	1273	Lactate dehydrogenase	+++	+++	H,E	A
**Additional Genes Identified by Microarray Analysis**						
*malR2*	0023	Transcriptional regulator, LacI family	++	−	−	R
*cldR*	0105	Cellodextrin LacI Transcriptional regulator	−	−	−	−
*#cldE*	0106	Cellodextrin permease	−	++	−	R
*glgP2*	0845	Glycogen phosphorylase	−	−	−	−
*rbsA1*	1419	Ribose ABC transporter, ATP binding protein	−	++	−	R
Bbr_1420	1420	Transcriptional regulator for Ribose utilisation, LacI family	−	+	−	−
Bbr_1658	1658	Sugar ABC transporter, solute binding protein	−	−	−	−
Bbr_1659	1659	Transcriptional regulator for Beta-glucoside utilization, LacI family	−	−	−	−
Bbr_1841	1841	ABC transporter, ATP binding protein	+	−	−	−
*gntR*	1891	Transcriptional regulator, GntR family	−	−	−	−
Bbr_1894	1894	PTS uptake system	++	+	H	−
*nrdH*	1901	Glutaredoxin	++	−	E	−

All reactions contain 150 nM protein (AraQ /MalR1) incubated with 0.5 nM Ird-labelled DNA fragments encompassing the promoter region of the specified gene. Binding affinity was calculated based on the total percentage DNA bound, – no binding, +binds up to 15%, ++ binds 15–50% and +++ binds 50–100% of DNA present in the reaction. *represents divergently orientated genes. ^♯^represents genes located at divergently orientated genes where the TSS has been experimentally validated.

Six of the genes/gene clusters whose promoter regions were clearly shown to be bound by AraQ and MalR1 encode enzymes that are part of the bifid shunt as illustrated in the schematic representation of central metabolism in Fig. 3. Several of these genes are essential for growth on glucose^43^ (Table 1) and are also highly transcribed during logarithmic growth on glucose as a sole carbon source^44^. The ability of AraQ to bind to the promoter regions of these six genes/gene clusters involved in central metabolism as well as those of certain genes involved in the metabolism of starch-like/-derived carbohydrates is consistent with previous *in silico* predictions^40^. EMSA analyses furthermore demonstrated that, similar to AraQ, MalR1 binds to promoter regions of genes/gene clusters involved in central carbon metabolism along with genes involved in the metabolism of starch-like/derived carbohydrates (and that the deduced AraQ and MalR1 regulons therefore largely overlap). The ability of MalR1 to transcriptionally control genes that are required for the metabolism of starch-like/-derived carbohydrates is consistent with *in silico* predictions^40^. However, MalR1’s presumed ability to control genes involved in central carbon metabolism was an unexpected finding, as these genes were not predicted to be members of MalR1’s predicted regulon^40^.

Based on their binding behaviour AraQ and MalR1 appear to have a similar affinity for the six promoter regions of the central metabolism genes (Table 1 and Fig. 3). Furthermore, AraQ and MalR1 were both found to be capable of binding their own and each other’s presumed promoter regions. This finding suggests that these genes are subject to interactive autoregulation, in which they not only control the transcription of their own genes, but also that of the other. Furthermore, as based on their binding behaviour, AraQ and MalR1 appear to control transcription of the LacI-type-encoding genes MalR2, MalR3, MalR5, MalR6 and CarD-like TF (Table 1).

### Determination of the operator sequences recognized by AraQ and MalR1

As shown above, EMSA analyses employing AraQ and MalR1 clearly demonstrate their *in-vitro* affinity for various promoter regions, including those that correspond to several central metabolic genes. In order to identify the TFBSs that are recognized by either AraQ or MalR1, fragmentation analysis was carried out (Fig. 1). This analysis was performed employing the promoter regions of the following genes: *eno* (encoding enolase and corresponding to locus tag Bbr_0725) and *pyk* (encoding pyruvate kinase and corresponding to locus tag Bbr_0757). These genes were selected as they which hold key roles in central carbon metabolism (Fig. 3). DNA fragments encompassing various sections of the promoter region upstream of either *eno* or *pyk* were subjected to EMSA analysis employing purified AraQ or MalR1. These analyses revealed that a 47 and a 48-bp DNA segment of the *eno* and *pyk* promoter regions, respectively, each harbour an operator site for both AraQ and MalR1 (Fig. 1). Importantly, both of these segments contain previously predicted candidate AraQ binding sites^40^, specifically the 47-bp segment of DNA located upstream of the *eno* gene contains a 20 bp imperfect palindrome CATTGTGAGC><GCTCACATCA (with “><” indicating the center of the palindrome), while the 48-bp DNA segment located upstream of the *pyk* gene contains a very similar 20 bp palindrome CAGTGTGAGC><GCTCACAACA.

Next, bioinformatic analysis through the use of a Position Weight Matrix (PWM) approach was employed to identify additional genes which may be regulated by AraQ and MalR1 in *B*. *breve*. For this task two PWM’s were used (see Materials and Methods section), resulting in the identification of several additional genes/gene clusters that appear to contain this TFBS in their predicted promoter region (though this will require experimental verification). The full list of genes assigned to the AraQ/MalR1 regulon is present in Supplemental Table S1. The majority of these regulated genes are involved in central carbohydrate metabolism and were previously classified as components of the global AraQ regulon in *Bifidobacteriaceae*^45^. Some *B*. *breve*-specific genes of the AraQ/MalR1 regulon include Bbr_0037/0038 (predicted anhydrase/alkyl hydroperoxide reductase), Bbr_0221-Bbr_0222 (iron-uptake system BfeU-BfeO)^46,47^, Bbr_0603 (predicted cholate transporter Ctr), Bbr_1316 and Bbr_1685 (hypothetical MFS transporters) (Supplemental Table S1). The use of a modified PWM which was designed based only on sequences of promoter regions with which AraQ/MalR1 had a medium or high affinity did not significantly change the regulon composition. Specifically, additional potential TFBSs were found in (intergenic) promoter regions of Bbr_0117/Bbr_0118 (glucosidase/sugar-binding protein), Bbr_0176 (penicillin binding protein), and Bbr_0921 (long-chain fatty acid-CoA ligase) (Supplemental Table S1; the derived AraQ/MalR1 binding motifs as based on these predicted sites are presented in Fig. 1, Panel 3).

Some divergence was identified between AraQ and MalR1 specificity for certain TFBSs, although for both TFs the affinity for these is rather low (Table 1). The construction of a PWM specific for either AraQ or MalR1 was not possible, the primary reason for this is that TFBSs of differentially bound genes contribute significantly less to the overall weight matrix than those associated with central metabolism (such as *eno* and *pyk*), which form the core of the regulon. Furthermore, this analysis found that AraQ and MalR1 bind with the highest affinity to promoter regions of genes involved in central metabolism. This observed high affinity may be explained by the conserved G, C, and A in positions 9, 12 and 15, respectively, of the AraQ/MalR1 binding motif (Fig. 1, Panel 3).

Interestingly, the deduced TFBSs of AraQ and MalR1 are located upstream of recently assigned −10/−35 promoter sites of six bifid shunt-associated genes/gene clusters (Table 1 and Supplemental Table S3)^24^. This TFBS location relative to the −10/−35 sites is therefore typical of a transcriptional activator and in this context it should be noted that all genes encoding the bifid shunt enzymes exhibit a high level of transcription when grown on glucose^24,44^. Further analysis showed that the TFBSs of AraQ/MalR1 are not located at a particular distance from these promoter sequences: Supplemental Table S3 illustrates that the AraQ/MalR1 TFBS is located 16 and 15 bp upstream of the −35 site in the promoter regions of *eno* and *pyk*, and 33, 34 and 35 bp upstream of the −35 site of *pfl*, *icfA* and *gap*, respectively. Therefore, the position of the AraQ/MalR1 TFBS relative to the −35 site implies that AraQ/MalR1 bind in multiples of one helical turn plus one half (i.e. 1.5, 3.5 or 6.5 helical turns) away from the transcriptional start sites (TSS). Finally, analysis of the deduced TSS with respect to the location of the TFBSs of AraQ/MalR1 upstream of *carD*, *glgP* and *cldE* revealed that the TFBS is overlapping with predicted −35/−10 regions, indicating that AraQ/MalR1 in these cases act as repressors, being more typical of LacI-type TFs^48^.

### Attempts to identify effector molecules for AraQ and MalR1

Bioinformatic and EMSA analyses allowed the deduction of the AraQ/MalR1 regulons and associated operator sequences. We next wanted to gain further insight into the nature of the environmental/metabolic signals that impact on the recognition ability and/or binding affinity of AraQ and MalR1 to their target DNA sequences. For this purpose, EMSA analyses were performed whereby AraQ or MalR1 was incubated with one of its binding targets, in this case the *araQ* DNA promoter region, in the presence of each of a variety of possible (available/relevant) effectors (Supplemental Table S4). These effectors were selected for two main reasons: (i) they occupy key positions within the bifid shunt (e.g. Phosphoenolpyruvate, D-erythrose-4-Phosphate and Glyceraldehyde 3-phosphate), which may allow them to act as cues to provide information on the metabolic status of the cell, and (ii) they are known to act as effector molecules in other organisms^45,49^. The results obtained indicate that under the employed experimental conditions none of the effector molecules tested in this study consistently decreased/increased AraQ or MalR1 ability to bind to DNA fragment encompassing the *araQ* promoter region (example effector EMSA can be found in Supplemental Fig. S2). It is possible that the *in vitro* experimental set up did not allow for the identification of the effector molecule(s), or perhaps that the molecule is the product of another metabolic pathway outside of central carbon metabolism.

### Transcriptome analysis of *B. breve* UCC2003-araQ and *B. breve* UCC2003-malR1

In order to further investigate the role of *araQ* and *malR1* in controlling central carbon metabolism, insertional mutants were created in each of these genes in *B*. *breve* UCC2003, resulting in strains *B*. *breve* UCC2003-*araQ* and *B*. *breve* UCC2003-*malR1* (respectively; see Materials and Methods). These two mutants were then employed to assess transcriptional changes due to either of these mutations using microarray analysis.

The transcriptome of *B*. *breve* UCC2003-*araQ*, when compared to that of *B*. *breve* UCC2003 (Supplemental Tables S5 and S6), revealed that 32 genes were transcriptionally up-regulated and 45 were down-regulated above a fold change of 2.0, p-value < 0.001 (see materials and methods for further details). Furthermore microarray data revealed that, when compared to *B*. *breve* UCC2003, the previously characterised malto-oligosaccharide uptake system (Bbr_0118- Bbr_0121)^50^ is significantly up-regulated in *B*. *breve* UCC2003-*araQ* (4.4, 4.4, 4.5 and 3.9-fold change respectively; p < 0.001). *In silico* analysis via a PWM-based approach identified AraQ/MalR1 TFBSs upstream of the above-mentioned gene cluster, the location of these TFBSs indicates that AraQ/MalR1 act as repressors of the Bbr_0118-Bbr_0121 gene cluster. Similarly, *malR3* (Bbr_0122; specifying a LacI type transcriptional regulator) and *apuB* (Bbr_0123; encoding an amylopullulanase) were up regulated 3.7 and 4.3-fold, respectively (p < 0.001) in *B*. *breve* UCC2003-*araQ* as compared with the wild type. *rbsD* (Bbr_1416), encoding a D-ribose pyranase, which is located in a ribose uptake cluster was also upregulated 2-fold (p < 0.001), PWM approach also identified the AraQ/MalR1 TFBS upstream of this cluster.

Most bifid shunt-associated genes whose promoter regions contain binding sites for AraQ/MalR1 were not significantly altered in the transcriptional analysis employing *B*. *breve* UCC2003-*araQ* or *B*. *breve* UCC2003-*malR1* (when compared to the transcriptome of wild type *B*. *breve* UCC2003; Supplemental Tables S5 and S6). This may be due to compensatory activity of each of the two regulators, in which AraQ will functionally compensate for loss of MalR1 (in *B*. *breve* UCC2003-*malR1*) and *vice versa* (for strain *B*. *breve* UCC2003-*araQ*). Attempts to create a double mutant of both *araQ* and *malR1* were unsuccessful, indicating that these two regulators form an essential transcriptional circuit of the bifid shunt genes.

The transcriptome of *B*. *breve* UCC2003-*malR1* revealed, when compared to that of the *B*. *breve* UCC2003 WT (Supplemental Tables S7 and S8), just a single gene, *malE1* (Bbr_0118), to be transcriptionally up-regulated above a fold change of 2.0, while no genes were found to be significantly (fold change of >2.0) down-regulated in the transcriptomic analysis of the *B*. *breve* UCC2003-*malR1* mutant. *In silico* analysis via a PWM-based approach identified a MalR1 TFBS upstream of *malE1* (Bbr_0118), where the location of this TFBS indicates that MalR1 acts as a repressor of this gene. Two genes, *apuB* and *malG1*, that form part of the predicted *malR1*-governed regulon^40^, were up-regulated to a lower, yet significant degree (1.8 and 1.3-fold change, respectively; P < 0.001). Notably, some of the central metabolic genes were found to be up-regulated at a rather modest, yet statistically significant level (i.e. *xfp*, *eno*, *tkt*, *pfl* and *ldh*, whose transcription was determined to be increased by 1.5, 1.3, 1.3, 1.2 and 1.2-fold, respectively; p-values < 0.004).

### Distribution of araQ and malR1 homologues among Bifidobacteriaceae members

In order to estimate conservation of the proposed AraQ/MalR1-mediated regulatory circuit in other bifidobacterial species and their close relatives, we analysed the distribution of the *araQ* and *malR1* genes in a set of 70 *Bifidobacteriaceae* genomes. This was carried out by aligning the 70 *Bifidobacteriaceae* genomes using a PATtyfam approach and the BLAST tool, followed by alignment with MUSCLE (see materials and methods for more details). The two regulator-encoding genes showed somewhat different patterns of distribution; *araQ* was fully conserved among all currently known species of the genus *Bifidobacterium*, while missing in genomes of closely related genera such as *Scardovia* and *Pseudoscardovia* (overall occurrence of *araQ* was determined to be 94%), whereas *malR1* was conserved in 50 genomes (overall occurrence 71%) (Supplemental Table S9). Interestingly, the absence of *araQ* in several *Scardovia* and *Pseudoscardovia* genomes coincides with the absence the arabinose utilization operon *araBDA*, which supports our previous hypothesis that AraQ evolved from a local regulator of arabinose metabolism in an ancestor *Bifidobacteriaceae* species to a global regulator of central carbohydrate metabolism in *Bifidobacterium* and *Gardnerella* by regulon expansion^51^.

A phylogenetic tree illustrating this information was also was built using IQ-TREE^52^ utilising an ultrafast bootstrap with 1000 replicates. BglR (Bbr_1659) is a distant LacI family regulator from *B*. *breve* UCC2003, this TF was employed as an out group as its function is predicted to be different from those of MalR1 and AraQ (Supplemental Fig. S3).

AraQ has been identified in distant lineages of Actinobacteria (functioning as a local TF)^45^, while orthologs of *malR1* (as well as the other *malR* genes) are confined to genera within the *Bifidobacteriaceae* family (Supplemental Table S9), implying that MalR1 is unique to this particular taxon and emerged after AraQ in the process of evolution. Although it is likely that MalR1 shares the same regulatory function as AraQ in many bifidobacteria by recognizing the same binding motif, this consideration is based on both the ubiquitous pattern of *malR1* distribution (especially when compared with genes of other MalR regulators, which are mosaically distributed among analysed strains and exhibit considerably lower prevalence), along with experimental validation through EMSA analysis. Thus, the possibility that MalR1 operates differently in other bifidobacteria or closely related genera cannot be entirely excluded. We were also unable to identify conserved joint AraQ/MalR1 binding sites in *Gardnerella vaginalis* ATCC 14019 using a PWM approach, the only analysed strain that lacks *araQ* yet possesses *malR1*. This observation suggests that in this bacterium MalR1 recognizes a different binding motif, and presumably works as a local TF regulating maltose/maltodextrin utilization.

### Survival of B. breve UCC2003-araQ and B. breve UCC2003-malR1 when exposed to porcine bile

**Figure 4:**
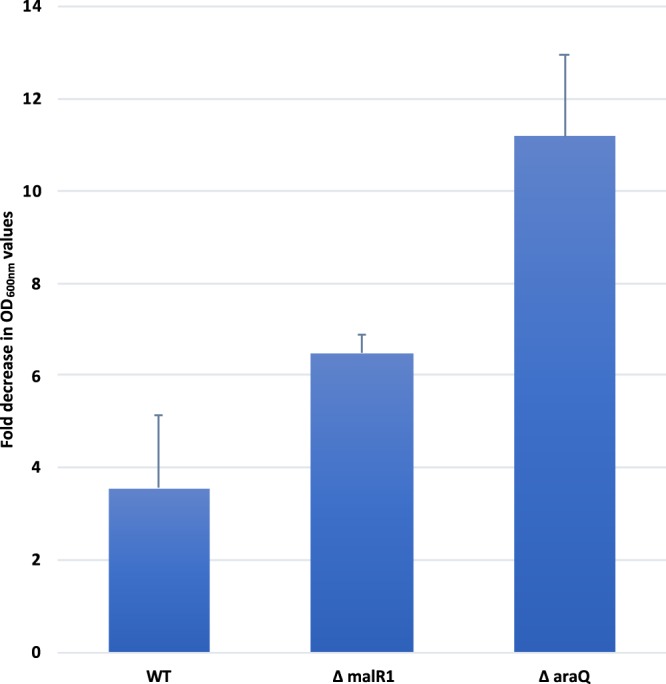
Phenotypic Growth Assay. Analysis of the growth of *B*. *breve* UCC2003 WT (WT), *B*. *breve* UCC2003-*araQ* (Δ*araQ*) and *B*. *breve* UCC2003-*malR1* (Δ*malR1*) when grown in the presence of 1% porcine bile. The values are expressed as fold decrease in OD_600nm_ values between growth on 0 and 1% bile. Error bars for each represent the standard deviation calculated from three replicates.

Several publications have reported that the (in)ability to survive bile stress is linked with shifts in the expression of genes involved in central metabolism and with the concentration of its resulting end products in various *Bifidobacterium* species^53–56^. For this reason, *B*. *breve* UCC2003 WT, *B*. *breve* UCC2003-*araQ* and *B*. *breve* UCC2003-*malR1* mutants were analysed for their ability to grow in the absence (control) as compared to the presence of 1% porcine bile with OD_600nm_ readings taken at 24-hour post inoculation. Figure 4 displays the fold reduction in OD_600nm_ following 24-hour growth in the presence of 1% porcine bile as compared to the control. A significant fold reduction in OD_600nm_ readings was observed for all three strains when grown in the presence of porcine bile, as compared to OD_600nm_ readings without porcine bile. However, the fold reduction in growth of *B*. *breve* UCC2003-*araQ* and *B*. *breve* UCC2003-*malR1* mutants was more pronounced compared to the wild type strain *B*. *breve* UCC2003: in the presence of 1% porcine bile, the *B*. *breve* UCC2003-*araQ* and *B*. *breve* UCC2003-*malR1* mutants demonstrated a fold reduction of 11.1 (*p*-value < 0.010) and 6.4 (*p*-value < 0.010) in OD_600nm_ readings, respectively, whereas the fold reduction for the control strain *B*. *breve* UCC2003 was 3.5.

## Discussion

The distal gut does not supply bacteria with a reliable carbon source; available carbohydrates are dependent on many factors such as host diet and other (competing) bacteria present in the GIT. Therefore, it is necessary for bifidobacteria to be capable of utilising a multitude of carbon sources and to do so efficiently in order to be competitive. Gene regulation is one way in which bifidobacteria ensure that their energy generation is in line with available carbohydrate substrates, and that energy and biosynthetic demands are kept in balance.

Previous analysis of the AraQ regulatory network in 39 bifidobacterial strains predicted that the conserved core of its regulon comprises genes involved in central carbohydrate metabolism^45^. In the current study, we experimentally validated these predictions. Additionally, it appears that AraQ and MalR1 together impose global shifts in gene transcription in *B*. *breve* UCC2003 through their apparent ability to regulate a number of TFs (i.e. CarD, MalR2, MalR3 and MalR5) and consequently the regulons of these TFs. AraQ and MalR1 were also found to control the transcription of key genes of the bifid shunt in *B*. *breve* UCC2003 (Fig. 3). Furthermore, AraQ and MalR1 appear to regulate transcription of their own genes (auto-regulation), while also regulating each other’s transcription. This mechanism of regulating central carbon metabolism with two LacI-type TFs is reminiscent of that of a previously described system in *Corynebacterium glutamicum*^57,58^, which utilises GntR1 and GntR2 to regulate many of its genes involved in central metabolism. From the current analysis AraQ and MalR1 are deduced to repress transcription of a number of genes related to sugar uptake, and to activate transcription of genes related to central carbon metabolism, while the opposite was seen for GntR1 and GntR2^58^. Initially, we believed that the functional redundancy of *malR1* was the product of a gene duplication event, as was predicted for *gntR1* and *gntR2*^58^. However, GntR1 and GntR2 exhibit 78% amino acid similarity, while AraQ and MalR1 display just 26% similarity. A higher level of similarity would be more in line with a (recent) gene duplication event. Additionally, it is also apparent from the phylogenetic analysis that AraQ and MalR1 form two non-orthologous groups (Fig. S3).

Analysis of *araQ* and *malR1* distribution across a set of 70 *Bifidobacteriaceae* genomes revealed slightly different patterns of distribution with *araQ* present in 94% and *malR1* present in 71% of these genomes tested. This distribution may be explained in a number of ways, with one possible evolutionary scenario being motif switching whereby MalR1 evolved to recognise the binding motif of AraQ. While duplicating the role of AraQ, the function of a maltose/maltodextrin utilization regulator, in that case, may have been transferred to other MalR paralogs. Additionally, it seems that in some bifidobacteria of insect/bird/marmoset origin *malR1* was lost probably due to different selective pressures experienced by these microorganisms. A PWM approach was also utilised to identify to identify additional genes which may be regulated by AraQ and MalR1 in *B*. *breve*. In some cases, AraQ/MalR1 PWMs failed to identify TFBSs of genes not involved in central metabolism to which recombinant TFs showed weak or medium affinity. This contradiction can be explained either by: (i) limitations of the EMSA approach, namely, non-specific binding of AraQ/MalR1 to sites of other TFs from the LacI family present in promoter regions of these genes; (ii) limitations of the PWM approach, specifically insufficient sensitivity of designed matrices.

The binding sites of AraQ/MalR1, where AraQ/MalR1 are believed to function as activators, were found to bind at a distance of a multiple of one turn plus one half, away from the TSS. This is consistent with findings for another LacI-type family member, CcpA, which may act as a repressor or activator^48^, and which binds 14 bp upstream of the −35 site when functioning as an activator. These authors suggested that CcpA binds to the same side of the helix face as the position of the −35 site. The AraQ/MalR1 TFBS was found (in some cases) to be located at a similar distance upstream of the −35 site implying that AraQ/MalR1 may also bind on the same side of the helix face as the position of the −35 site. One important feature of LacI-type TFs is that they can alter the local DNA structure^48^, which in this case may enhance the affinity of the RNA polymerase holoenzyme to its cognate −35/−10 sites.

Transcriptional analysis for *B*. *breve* UCC2003-*araQ* vs the *B*. *breve* UCC2003 WT strain demonstrated that transcription of a number of genes is repressed by AraQ, whereas genes where AraQ is predicted to act as a transcriptional activator (based on the location of the TFBS in relation to the −35 site) showed no significant change in expression. This result is in line with our predictions as insertional mutagenesis should reduce the amount of active AraQ protein in the cell, therefore the expression of those genes which AraQ activates should remain unchanged.

The biological significance of a bacterium producing two distinct proteins which appear to have overlapping functions is currently unknown. We hypothesize that there may be a link between the presence of these two proteins and the stress response of the bacterium. Several publications have reported that the (in)ability to survive bile stress is linked with shifts in the expression of genes involved in central metabolism and with the concentration of its resulting end products in various *Bifidobacterium* species^53–56^. For this reason, growth assays were carried out on *B*. *breve* UCC2003-*araQ* and *B*. *breve* UCC2003-*malR1*, along with the wild type control strain *B*. *breve* UCC2003, when exposed to porcine bile (Fig. 4). This analysis revealed that there is a statistically significant drop in viable cell count for both *B*. *breve* UCC2003-*araQ* and *B*. *breve* UCC2003-*malR1*, suggesting that this hypothesis may be correct yet requires further in-depth investigation. The mechanism behind this phenotype remains unclear, though one possible cause may be that *B*. *breve* UCC2003-*araQ* is unable to quickly adjust its central metabolism in an optimal way under this stress condition, as previously reported^59^. AraQ and MalR1 binding sites have also been identified upstream of a predicted choline transporter (Bbr_0603) and an iron uptake system (Bbr_0121-0122)^46,47^, indicating that these TFs control a number of genes involved in bile and other cell stress responses. AraQ and MalR1 ability to control central metabolism may also aid survival under bile stress conditions by increasing ATP production from the bifid shunt, while fine tuning the expression on essential central metabolic genes, a role which would benefit from the presence of two distinct TF proteins.

As with all *in vitro* assays, EMSA and transcriptomic analyses have their limitations and it is important to consider these factors on interpretation of the obtained results. Nonetheless, our findings indicate that AraQ and MalR1 play an important role in regulating the metabolic flux of central metabolism in a large proportion of the *Bifidobacteriaceae*, presumably contributing to metabolic agility and associated ability to adapt to stressful conditions. In conclusion, this work revealed a novel and apparently unique regulatory mechanism employed by most human-derived bifidobacteria. This regulatory system appears to be crucial to provide adaptability and competitiveness in the constantly changing environment of the gastrointestinal tract.

## Methods

### Bacterial strains and culture conditions

Bacterial strains and plasmids are listed in Table 2. *B*. *breve* strains were routinely grown at 37 °C in either de Man Rogosa and Sharpe medium (MRS medium; Difco, BD, Le Pont de Claix, France), modified de Man Rogosa and Sharpe (mMRS) medium made from first principles^60^, or reinforced clostridial medium (RCM; Oxoid Ltd., Basingstoke, Hampshire, UK) supplemented with 0.05% cysteine-HCl. Bifidobacteria were incubated anaerobically in a modular, atmosphere-controlled system (Davidson and Hardy, Belfast, Ireland). *Escherichia coli* strains were routinely grown in Luria Bertani (LB) broth at 37 °C with liquid cultures agitated at 120 rpm. Where appropriate growth medium was supplemented with tetracycline (Tet; 10 µg ml^−1^), chloramphenicol (Cm; 5 µg ml^−1^ for *E*. *coli*, 2.5 µg ml^−1^ for *B*. *breve*), ampicillin (Amp; 100 μg ml^−1^), erythromycin (Em; 100 µg ml^−1^) or kanamycin (Kan; 50 µg ml^−1^) for plasmid/strain selection and/or maintenance.

**Table 2:** Bacterial strains and plasmids used in this study. Em^r^, Km^r^, Tet^r^ and Amp^r^: resistance to erythromycin, kanamycin, tetracycline and ampicillin, respectively.

Strains and plasmids	Relevant features	Reference or source
**Strains**		
***B***. ***breve***		
UCC2003	Isolate from nursling stool	^80^
UCC2003-*malR1*	pORI19-*tetW*-*malR1* insertion mutant of UCC2003	This study
UCC2003-*araQ*	pORI19-*tetW*-araQ insertion mutant of UCC2003	This study
***E***. ***coli***		
XL1-BLUE	Host for pQE60 plasmids; supE44 hsdR17 recA1 gyrA96 thi relA1 lac F = [proAB laclq lacZ M15 Tn10(Tet^r^)]	Stratagene
XL1-BLUE + pQE60	pQE60 *E*. *coli* expression vector, Amp^r^	This study
EC101 + PQE60_*malR1*	pQE60 + *malR1*	This study
XL1-BLUE + pQE60_*araQ*	pQE60 + *araQ*	This study
EC101	Cloning host for pORI19 for insertional mutagenesis; *repA*^+^ *Km*^*r*^ *and* pQE60 *E*. *coli* expression vector	^42^
**Plasmids**		
pORI19	Em^r^, repA^−^, ori^+^, cloning vector	^42^
pORI19-*malR1*	pOR19 harbouring internal fragment of *malR1* (*Bbr_1846*)	This study
pORI19-*araQ*	pOR19 harbouring internal fragment of *araQ* (*Bbr_0411*)	This study
pORI19-*malR1*-tet	pOR19 harbouring internal fragment of *malR1* (*Bbr_1846*) *+* Tet^r^	This study
pORI19-*araQ*-tet	pOR19 harbouring internal fragment of *araQ* (*Bbr_0411*) + Tet^r^	This study
pQE60	*E*. *coli* expression vector, Amp^r^	Qiagen
pQE60 + malR1	pQE60 harbouring *malR1*	This study
pQE60 + araQ	pQE60 harbouring *araQ*	This study

### Nucleotide sequence analysis

Sequence data were obtained from the Artemis-mediated^61^ genome annotations of the *B*. *breve* UCC2003 genome sequence^62^. Database searches were carried out using non-redundant sequences accessible at the National Centre for Biotechnology Information internet site (http://www.ncbi.mlm.nih.gov) utilising the basic local alignment search tool (BLAST). Sequence analysis was performed employing the Seqbuilder and Seqman programs of the DNASTAR software package (DNASTAR, Madison, WI). Protein functions were assigned with the use of BLASTP, and homology detection and structure prediction by HMM-HMM comparison and HHpred^63,64^.

For comparative studies, a non-redundant set of 70 *Bifidobacteriaceae* genomes was created. The search for AraQ and MalR1 orthologs in these genomes was carried out using the PATtyfam approach and the BLAST tool integrated into the SEED genomic platform^65^. Sequences of orthologs were collected into one file and then aligned via MUSCLE^66^. The alignment was subsequently manually filtered to reduce the number of gaps. A phylogenetic tree was built using IQ-TREE^52^ with the following parameters: automatic substitution model selection, ultrafast bootstrap with 1000 replicates, and employing BglR (Bbr_1659), a distant LacI family regulator from *B*. *breve* UCC2003 with predicted different function, as an outgroup.

### DNA manipulations

DNA manipulations were carried out based on previously published protocols^67^. Restriction enzymes and T4 DNA ligase were obtained from Roche Diagnostics (Basel, Switzerland), and were used according to the manufacturer’s instructions. PCRs were performed using either Q5® High-Fidelity DNA polymerase (New England Biolabs, Hertfordshire, UK) or Extensor Long Range PCR Enzyme master mix (Thermo Scientific, Glouchester, UK). Synthetic oligonucleotides were synthesized by Eurofins (Ebersberg, Germany) and are listed in supplemental Table S2 and S10. Ird-labelled synthetic oligonucleotides were provided by IDT (Integrated DNA technologies, Dresden, Germany) and are listed in Supplemental Table S2. PCR products were purified with the use of a High-Pure PCR product purification kit (Roche, Basel, Switzerland). Plasmid DNA was introduced into *E*. *coli* and *B*. *breve* by electroporation, and large-scale preparation of chromosomal DNA from *B*. *breve* was performed as described previously^68^. Plasmid DNA was obtained from *B*. *breve* and *E*. *coli* using the Roche High Pure plasmid isolation kit (Roche Diagnostics, Basel, Switzerland). An initial lysis step was performed using 30 mg ml^−1^ of lysozyme for 30 min at 37 °C as part of the plasmid purification protocol for *B*. *breve*.

### Construction of *B. breve* UCC2003 insertion mutants

Internal fragments of Bbr_0411 (designated here as *araQ*; fragment used was 402 bp in length, representing codons 74 through to 208 of the 371 codons of this gene) and Bbr_1846 (designated here as *malR1*; fragment used was 504 bp in length, representing codons 102 through to 267 of the 338 codons of this gene) were amplified by PCR using *B*. *breve* UCC2003 chromosomal DNA as a template (primers employed are listed in Supplemental Table S10). Insertional mutagenesis was carried out as previously described^69^. The presence of the tetracycline resistance cassette was confirmed by colony PCR employing primer combination TetF and TetR, while site-specific recombination of potential Tet-resistant mutants was confirmed by colony PCR using a combination of the TetR primer and a primer located upstream of the recombination site in *araQ* or *malR1* in the chromosome of *B*. *breve* UCC2003 (see Supplemental Table S10 for primer details). The confirmed insertional mutants within *araQ* and *malR1* were designated here as *B*. *breve* UCC2003-*araQ* and *B*. *breve* UCC2003-*malR1*, respectively.

### Microarray analysis

The transcriptome of *B*. *breve* UCC2003-*araQ* and *B*. *breve* UCC2003-*malR1* was compared to the global gene expression patterns of *B*. *breve* UCC2003 (WT). Insertional mutants and the WT strain were cultivated in mMRS medium supplemented with 0.5% ribose until an OD_600nm_ of ∼0.6 was achieved. We employed ribose for such transcriptome analyses as this sugar has been utilized previously as a reference carbohydrate for various metabolic studies^37,70–72^. Furthermore, transcriptome analysis for *B*. *breve* UCC2003-*araQ* and *B*. *breve* UCC2003 WT was carried out when these strains were grown on lactose as a sole carbon source, which generated similar results as compared to ribose-grown cells except for the expected sugar-specific differences. Cells were harvested by centrifugation at 10,000 rpm for 2 min at room temperature and immediately frozen at −80 °C prior to RNA isolation. DNA microarrays containing oligonucleotide primers representing each of the annotated genes on the genome of *B*. *breve* UCC2003 were designed by and obtained from Agilent Technologies (Palo Alto, CA, USA). Cell disruption, RNA isolation, RNA quality control, and cDNA synthesis and labelling were performed as described previously^73^. The labelled cDNA was hybridized using the Agilent Gene Expression hybridization kit (part number 5188-5242) as described in the Agilent Two-ColorMicroarrayBased Gene Expression Analysis v4.0 manual (G4140-90050).

Following hybridization, the microarrays were washed in accordance with Agilent’s standard procedures and scanned using an Agilent DNA microarray scanner (model G2565A). The generated scans were converted to data files with Agilent’s Feature Extraction software (v9.5). The DNA microarray data sets were processed as previously described^53,74,75^. Differential expression tests were performed with the Cyber-T implementation of a variant of the t-test^76^. A gene was considered to exhibit a significantly different transcription level relative to the control when p < 0.001 and an expression ratio of >2 or <0.25. The microarray data sets obtained in this study have been deposited in NCBI’s Gene Expression Omnibus database and are accessible through GEO series accession number GSE108949.

### AraQ and MalR1 expression and purification

For the construction of plasmids pQE60 + *araQ* and pQE60 + *malR1*, DNA fragments encompassing *araQ* (corresponding to locus tag Bbr_0411) and *malR1* (corresponding to locus tag Bbr_1846) were generated by PCR amplification employing chromosomal DNA of *B*. *breve* UCC2003 as a template, Q5 high-fidelity DNA polymerase, and primers araQ_F and araQ_R, and malR1_F and malR1_R, respectively (Supplemental Table S2). An in-frame His10-encoding sequence is contained within the 3’ end of the pQE60 construct to facilitate downstream protein purification. The *araQ*-encompassing PCR product was digested with NcoI and BglII, while the *malR1*-encompassing PCR product was digested with NcoI and BamHI. The digested PCR products were ligated into a similarly digested pQE60, an IPTG-inducible translational fusion plasmid. The ligation mixtures were introduced into *E*. *coli* XL1-Blue or *E*. *coli* EC101 by electro-transformation, and transformants were then selected on the basis of Ampicillin resistance (Amp^R^). The plasmid contents of a number of Amp^R^ transformants were screened by restriction analysis, and the integrity of positively identified clones was verified by sequencing. One verified clone of plasmid pQE60 + *araQ* and pQE60 + *malR1* (i.e. plasmid pQE60 in which either *araQ* or *malR1*, respectively, was cloned) (Table 2) was then selected for protein expression and purification purposes.

*E*. *coli* XL1-BLUE was utilised for heterologous expression of AraQ and MalR1. *E*. *coli* XL1-BLUE strains containing either pQE60 + *araQ* or pQE60 + *malR1* were inoculated at 2% in LB medium and grown until an OD_600nm_ of ∼0.5 was reached, at which point protein expression was induced by the addition of IPTG^25^. Following incubation for a further two hours cells were harvested by centrifugation and re-suspended in EMSA buffer (see below). Bacterial cells were disrupted by bead beating in a mini-bead beater (BioSpec Products, Bartlesville, OK, USA) using glass beads. Cellular debris was removed by centrifugation to produce a crude cell extract. Recombinant AraQ and MalR1 proteins, each tagged with an incorporated C-terminal His_10_ sequence, were purified from a crude cell extract using a nickel-nitrilotriacetic acid column (Qiagen, Hilden, Germany) according to the manufacturer’s instructions (QIAexpressionist, June 2003). Lysis, wash and elution buffers were supplemented with 10% glycerol as this considerably improved the (binding) activity of AraQ and MalR1. Elution fractions were analysed by SDS-polyacrylamide gel electrophoresis, as described previously^77^, on a 12.5% polyacrylamide gel. Following electrophoresis, gels were fixed and stained with Coomassie brilliant blue to identify fractions containing the purified protein. Colour Prestained Protein Standard, Broad Range (11–245 kDa) (New England BioLabs, Hertfordshire, UK) was used to estimate the molecular weights of the purified proteins. Protein was concentrated using Amicon® Ultra Filters from Merck Millipore and dialysed into EMSA binding buffer (80 mM Tris-HCl [pH 8.0], 20 mM MgCl_2_, 2 mM dithiothreitol [DTT], 4 mM EDTA, 400 mM KCl and 40% glycerol). Concentration of the purified AraQ/MalR1 protein and subsequent dialysis into EMSA binding buffer also improved the binding ability of these two LacI-type proteins. Protein concentrations were determined using the Qubit® fluorometer as per manufacturer’s instructions (Thermofisher scientific, Glouchester, UK). Purified protein was aliquoted and stored at -80 °C for subsequent use in EMSAs.

### Electrophoretic mobility shift assay (EMSA)

Promoter regions of genes of interest were amplified by PCR utilising individual primer pairs, of which one or both were 5’ Ird-700-labelled (provided by IDT, Dresden, Germany) as listed in supplemental Table S3. Electrophoretic mobility shift assays (EMSAs) were performed as described previously^78^. All binding reactions were carried out with poly(dI-dC) (0.05 µg µl^−1^), DNA probe (0.5 nmol), BSA (0.2 µg µl^−1^), binding buffer (80 mM Tris-HCl [pH 8.0], 20 mM MgCl_2_, 2 mM dithiothreitol [DTT], 4 mM EDTA, 400 mM KCl and 40% glycerol) and water to a final volume of 20 μl. Binding reactions were typically carried out with 150 nM purified AraQ or MalR1 and 0.5 nM Ird-700-labelled DNA, and incubated for 20 min at 37 °C prior to loading onto a 6% non-denaturing polyacrylamide (PAA) gel prepared in TAE buffer (40 mM Tris-acetate [pH 8.0], 2 mM EDTA) and bound/unbound DNA fragments were then separated by electrophoresis on a 0.5X to 2.0X gradient of TAE at 100 V for 90 min in an Atto Mini PAGE system (Atto Bioscience and Biotechnology, Tokyo, Japan).

Signals and percentage binding inferred from the Integrated Intensity (II) were detected/calculated using an Odyssey infrared imaging system (Li-Cor Biosciences, United Kingdom, Ltd., Cambridge, United Kingdom), and images were electronically captured with the use of Odyssey software v3.0. The ability of AraQ and MalR1 to bind DNA fragments was assessed employing an arbitrary, non-linear scale which spanned from no affinity, low, medium to high affinity (and annotated as −, +, ++, +++, respectively). This non-linear scale was correlated to the percentage of DNA which was bound as compared to unbound target DNA within a given reaction with – representing no DNA bound, + represents up to 15% DNA bound, ++ represents 15% – 50% DNA bound and +++ represents 50–100% DNA bound. In some cases, when a DNA fragment was shown to exhibit binding affinity, the binding site was more precisely determined by generating sub-fragments by PCR, followed by EMSAs as described above. To identify possible effectors for AraQ or MalR1, 10 mM of a particular compound (Supplemental Table S4) was added to the binding reaction mixture.

### Motif searches

Motif searches were carried out using Position Weight Matrix (PWM) approach as described previously^40^. Several PWMs were implemented: (i) a PWM used for the AraQ regulon reconstruction in bifidobacteria based on a previous study^40^; (ii) a new AraQ/MalR1 PWM which was built using the EMSA data as a training set (based on promoter regions with which AraQ/MalR1 had a medium affinity (++) or high affinity (+++). A graphical representation of the identified motifs was obtained using WebLOGO software^79^.

### Phenotypic analysis on bile

In order to assess the effects of bile on growth, *B*. *breve* UCC2003 and its isogenic derivatives *B*. *breve* UCC2003-araQ and *B*. *breve* UCC2003-malR1 were inoculated at 1% (v/v) from stock into mMRS medium supplemented with 1% maltose and 0.5% (v/v) L-cysteine HCl and were cultured overnight under anaerobic conditions at 37 °C. The above strains were then inoculated at 1% (v/v) into mMRS medium containing 1% maltose, 0.5% (v/v) L-cysteine HCl along with increasing concentration of porcine bile at 0, 1 and 2% (v/v). mMRS without the addition of a carbohydrate source served as a negative control. Bacteria were evaluated for their ability to grow in the presence of bile with Optical density (OD_600nm_) readings taken at 10 and 24 hr post-inoculation. Samples were assessed as biologically independent triplicates. Statistical analysis was carried out using the Microsoft Excel Data Analysis ToolPak. The statistical significance was determined for the two mutants, by comparing the difference in their OD_600nm_ readings as compared with the WT control when exposed to bile.

## Supplementary information

Supplementary Information

## Data Availability

All data generated and/or analysed during this study are included in this article and its Supplementary Information files.
