# Supplementary material for: Transcriptional control of central carbon metabolic flux in Bifidobacteria by two functionally similar, yet distinct LacI-type regulators
Source: Sci Rep. 2019 Nov 28;9:17851. doi: 10.1038/s41598-019-54229-4 (PMC6882875; doi:10.1038/s41598-019-54229-4)
Supplement: Supplementary file 1 — Supplementary Information [file 41598_2019_54229_MOESM1_ESM.pdf]

Transcriptional control of central carbon metabolic flux in Bifidobacteria by two functionally similar, yet distinct LacI-type regulators

Noreen Lanigan[1], Emer Kelly[1], Aleksandr A. Arzamasov[2,3], Catherine Stanton[4], Dmitry A Rodionov[2,3] and Douwe van Sinderen[1*].

[1] School of Microbiology & APC Microbiome Ireland, University College Cork, Ireland University College Cork, Cork, Ireland;
[2] Sanford Burnham Prebys Medical Discovery Institute, La Jolla, United States,
[3] A.A. Kharkevich Institute for Information Transmission Problems, Russian Academy of Sciences, Moscow, Russia
[4] Teagasc Food Research Centre, Moorepark, Fermoy, Cork, Ireland

Correspondence to [d.vansinderen@ucc.ie]

Supplemental Tables

Supplemental Table S1. Sites found using PWM From Khoroshkin et al, 2016 40

Sites found using PWM from Khoroshkin et al., 2016							
**Locus_tag**	**Gene/operon**	**Site**	**Pos***	**Score**	**Binding AraQ**	**Binding MalR1**	**Additional information**
Bbr_0032	*malR5*	aAaTGatAGCGCTtACAtTg	-264	4.59	+	+	AraQ regulon in Bifidobacteriaceae
		aAccGTGAcCGCTaACAAcg	-158	4.93			Also predicted as MalR5 sites
Bbr_0033	*malE2*	cgTTGTtAGCGgTCACggTt	-226	4.93			
		cAaTGTaAGCGCTatCAtTt	-120	4.59			
Bbr_0037	*icfA*	gATTGTGAGCGtTCtCAtTA	-115	5.13	NM	NM	
Bbr_0038	*ahpC*	TAaTGaGAaCGCTCACAATc	-173	5.13			
Bbr_0221	*bfeU - bfeO*	ggaTGTtAcCGCTCACAtgc	-221	4.82	NM	NM	
Bbr_0411	*araQ*	cAaTGTGAGCGCTCACgcgA	-112	5.38	+++	+++	AraQ regulon in Bifidobacteriaceae
Bbr_0603	*ctr*	acaTGcGAGCGtTCACgATg	-101	4.56	NM	NM	
Bbr_0725	*eno*	cATTGTGAGCGCTCACAtcA	-110	5.99	+++	+++	AraQ regulon in Bifidobacteriaceae
Bbr_0746	*glgB* - Bbr_745 - Bbr_744	cAaTGaGAGCGCTCACAATc	-108	5.3	+++	++	AraQ regulon in Bifidobacteriaceae
Bbr_0747	*carD - ispF*	gATTGTGAGCGCTCtCAtTg	-91	5.3			
Bbr_0757	*pyk*	cAgTGTGAGCGCTCACAAcA	-130	5.92	+++	+++	AraQ regulon in Bifidobacteriaceae
Bbr_0772	*pta - ackA*	cAccGTGAaCGaTaACAAcg	-76	4.59	NM	NM	AraQ regulon in Bifidobacteriaceae
Bbr_0787	*pflB - pflA*	gATTGgtAGCGCTCACAgaA	-135	5.27	++	+++	AraQ regulon in Bifidobacteriaceae
Bbr_1003	*tkt - tal*	aATTGTGAaCGCTaACAgaA	-156	5.68	++	++	AraQ regulon in Bifidobacteriaceae
Bbr_1233	*gap*	cAaTGTGAGCGCTCACAAaA	-156	6.02	+++	+++	AraQ regulon in Bifidobacteriaceae
Bbr_1237	*galM*	gATTGcGAGCGgTaACAATA	-46	4.74	NM	NM	AraQ regulon in Bifidobacteriaceae
Bbr_1273	*ldh2*	ggcTGTGAGCGCTaACAAct	-160	5.77	+++	+++	AraQ regulon in Bifidobacteriaceae
Bbr_1316	Bbr_1316	ctcTGTtAtCGtTCACAATg	-250	4.89	NM	NM	
Bbr_1419	*rbsA1 - rbsC1 - rbsB1 - rbsD1 - rbsK2*	gATTGTtAaCGCTCtCgAcA	-184	4.76	-	++	
Bbr_1650	*malQ2*	ccaTGTGAGCGtTCtCAtgt	-138	4.51	NM	NM	AraQ regulon in Bifidobacteriaceae
Bbr_1685	Bbr_1685	ctaTGTGAaCGCTCtCAAgc	-232	4.75	NM	NM	
Bbr_1723	*birA*	acgTGTGAGCGtTCACAccg	-107	5.2	NM	NM	AraQ regulon in Bifidobacteriaceae
Bbr_1724	Bbr_1724 - Bbr_1725	cggTGTGAaCGCTCACAcgt	-158	5.2	NM	NM	
Bbr_1847	*malE*	gcaTGTtAaCGCTCtCAtTg	-247	4.58	++	-	AraQ regulon in Bifidobacteriaceae
Sites found using new PWM							
Bbr_0032	*malR5*	aAATGatAGCGCTtACATTg	-264	5.14	+	+	AraQ regulon in Bifidobacteriaceae
		aAccGTGAcCGCTaACAacg	-158	4.64			Also predicted as MalR5 sites
		gAATGTaAGCGCatACATat	-119	4.65			
Bbr_0033	*malE2*	atATGTatGCGCTtACATTc	-265	4.65			
		cgtTGTtAGCGgTCACggTt	-226	4.64			
		cAATGTaAGCGCTatCATTt	-120	5.14			
Bbr_0037	*icfA*	gAtTGTGAGCGtTCtCATTA	-115	5.6	NM	NM	
Bbr_0038	*ahpC*	TAATGaGAaCGCTCACAaTc	-173	5.6			
Bbr_0117	*agl4*	TgATGcataCGCTCgCATTA	-179	4.61	++	++	Also predicted as MalR3 sites
Bbr_0118	*malE1*	TAATGcGAGCGtatgCATcA	-99	4.61			
Bbr_0176	*pbp2*	cccTGaGAGCGtTCACgTcA	-55	4.8	NM	NM	
Bbr_0221	*bfeU - bfeO*	ggATGTtAcCGCTCACATgc	-221	5	NM	NM	
Bbr_0411	*araQ*	cAATGTGAGCGCTCACgcgA	-112	5.53	+++	+++	AraQ regulon in Bifidobacteriaceae
Bbr_0603	*ctr*	acATGcGAGCGtTCACgaTg	-101	4.87	NM	NM	
Bbr_0704	Bbr_0704	TAATGcaAaCGtTagCATTA	-69	4.66	NM	NM	
Bbr_0725	*eno*	cAtTGTGAGCGCTCACATcA	-110	6.32	+++	+++	AraQ regulon in Bifidobacteriaceae
Bbr_0746	*glgB* - Bbr_745 - Bbr_744	cAATGaGAGCGCTCACAaTc	-108	5.8	+++	++	AraQ regulon in Bifidobacteriaceae
Bbr_0747	*carD - ispF*	gAtTGTGAGCGCTCtCATTg	-91	5.8			
Bbr_0757	*pyk*	cAgTGTGAGCGCTCACAacA	-130	6.12	+++	+++	AraQ regulon in Bifidobacteriaceae
Bbr_0772	*pta - ackA*	cAccGTGAaCGaTaACAacg	-76	4.51	NM	NM	AraQ regulon in Bifidobacteriaceae
Bbr_0787	*pflB - pflA*	gAtTGgtAGCGCTCACAgaA	-135	5.36	++	+++	AraQ regulon in Bifidobacteriaceae
Bbr_0921	*fadD2*	cgATGTGAaCGaTtACAaTc	-81	4.82	NM	NM	
Bbr_1003	*tkt - tal*	aAtTGTGAaCGCTaACAgaA	-156	5.45	++	++	AraQ regulon in Bifidobacteriaceae
Bbr_1233	*gap*	cAATGTGAGCGCTCACAaaA	-156	6.23	+++	+++	AraQ regulon in Bifidobacteriaceae
Bbr_1237	*galM*	gAtTGcGAGCGgTaACAaTA	-46	5.11	NM	NM	AraQ regulon in Bifidobacteriaceae
Bbr_1273	*ldh2*	ggcTGTGAGCGCTaACAact	-160	5.57	+++	+++	AraQ regulon in Bifidobacteriaceae
Bbr_1316	Bbr_1316	ctcTGTtAtCGtTCACAaTg	-250	4.88	NM	NM	
Bbr_1419	*rbsA1 - rbsC1 - rbsB1 - rbsD1 - rbsK2*	gAtTGTtAaCGCTCtCgacA	-184	4.83	-	++	
Bbr_1650	*malQ2*	ccATGTGAGCGtTCtCATgt	-138	5.02	NM	NM	AraQ regulon in Bifidobacteriaceae
Bbr_1685	Bbr_1685	ctATGTGAaCGCTCtCAagc	-232	5.12	NM	NM	
Bbr_1723	*birA*	acgTGTGAGCGtTCACAccg	-107	5.2	NM	NM	AraQ regulon in Bifidobacteriaceae
Bbr_1724	Bbr_1724 - Bbr_1725	cggTGTGAaCGCTCACAcgt	-158	5.2	NM	NM	
Bbr_1847	*malE*	gcATGTtAaCGCTCtCATTg	-247	5.1	++	-	AraQ regulon in Bifidobacteriaceae
Divergons are marked by thick box borders							
NM = not measured							
New members are in yellow (in comparison with old PWM)							
*Position of a site relative to the first nucleotide of CDS							

Supplemental Table S2. Oligonucleotide primers used in EMSA analysis.

The locus tag number refers to the gene upstream of which a presumed promoter region is present that was amplified by the primer pair. If two locus tags are indiciated, it means that the promoter region is located in between two corresponding and divergently oriented genes (i.e., representing an intergenic region). Primers used in the EMSA experiments, Ird represents primers which were labelled with an iridescent probe at the 5' end.

**Locus tag**	**Primer name**	**Sequence**
**Bbr_0023**	*malR2*_Ird	gctcgcttttgccatggc
**Bbr_0023**	*malR2*	ccgaaaccagccgttgcac
**Bbr_0027**	*malFG 2*_Ird	ccatgccggtctcctttgc
**Bbr_0027**	*malFG 2*	gccagcctactttctcctcc
**Bbr_0032/Bbr_0033**	*malR5/malE2*_Ird	cgccatgatgtctcctttcgc
**Bbr_0032/Bbr_0033**	*malR5/malE2*	gctcatgtgatacgtgcctcc
**Bbr_0060**	*glgP 1*_Ird	cattctgactttccttccggg
**Bbr_0060**	*glgP 1*	gcgttctatccttgcactgagcc
**Bbr_0105**	*cldR*_Ird	gctgcgcgctatgttctcct
**Bbr_0105**	*cldR*	ccacgtcacgaatagttgcc
**Bbr_0106**	*cldE*_Ird	gcgacgatgacgaatccg
**Bbr_0106**	*cldE*	gcacttgggcgctcattg
**Bbr_0111**	*agl3*_Ird	gttcattcagcccgacgc
**Bbr_0111**	*agl3*_Ird	gtcattgaggttggcg
**Bbr_0112/Bbr_0113**	*malR6/*Bbr_0113_Ird	cgcttgaatgcttgcttttgc
**Bbr_0112/Bbr_0113**	*malR6/*Bbr_0113	ccggaacttctcgctatcatcatg
**Bbr_0116**	*malQ 2*_Ird	cctgttctgctcttaccgtagctc
**Bbr_0116**	*malQ 2*	gagctacggtaagagcagaacagg
**Bbr_0117/Bbr_0118**	*agl4/malFG*_Ird	cggttcctacgccaagtaatc
**Bbr_0117/Bbr_0118**	*agl4/malFG*	aagtgctgctgctgtcatcg
**Bbr_0122/Bbr_0123**	*malR3/apuB*_Ird	ggtagatgtctgccttgccc
**Bbr_0122/Bbr_0123**	*malR3/apuB*_Ird	gcaaggcgtttggcgagcg
**Bbr_0411**	*araQ*_Ird	cgcacttcctggcatttg
**Bbr_0411**	*araQ*	gaacttcaggccgccagc
**Bbr_0725**	*eno*_Ird	caaggaagtcgccgacaatc
**Bbr_0725**	*eno*	ggttgccacgagaatccag
**Bbr_0747**	*carD*_Ird	ggcacattgatgaccagacc
**Bbr_0747**	*carD*_Ird	gccgttgtattcgtgtgtca
**Bbr_0757**	*pyk*_Ird	cgctgagaaggcctgaaatc
**Bbr_0757**	*pyk*_Ird	ggttgtcgtaatcctcggtg
**Bbr_0787**	*pflBA*_Ird	gccgatagaacagcgtatgg
**Bbr_0787**	*pflBA*	cttggcgtcgagctcctcttg
**Bbr_0845**	*glgp 2*_Ird	cgcacctccttccacgctg
**Bbr_0845**	*glgp 2*	ccgttagattggagattgtccg
**Bbr_1002-1003**	*tkt – tal*_Ird	gctcggtctccttgaattcg
**Bbr_1002-1003**	*tkt – tal*	ggctcttgcgaacgaatg
**Bbr_1233**	*gap*_Ird	gcattgcctcaggtaagcc
**Bbr_1233**	*gap*	gaccaatgcgaccgaagc
**Bbr_1273**	*ldh*_Ird	ggatgtcgattcgcacttgg
**Bbr_1273**	*ldh*_Ird	agcttgctgttattggtgcc
**Bbr_1419**	*rbsA1*_Ird	gctcaatagtccttcgccgcc
**Bbr_1419**	*rbsA1*	catacgcctctcgctttcgtc
**Bbr_1420**	*lacI*_Ird	gatcatgctcagatgcggcg
**Bbr_1420**	*lacI*	cgaatgccatatccgtctcc
**Bbr_1595**	*pgma*_Ird	ccatactttcattctgccacg
**Bbr_1595**	*pgma*	gcgacatctcttactccattcc
**Bbr_1658**	Bbr_1658_Ird	ggtcaagctcatcgtgcg
**Bbr_1658**	Bbr_1658	ctggtcagcatagccgcac
**Bbr_1659**	*LacI*_Ird	caacgtgcgcagcctatgg
**Bbr_1659**	*LacI*	cttcgccacgtcatacacc

**Bbr_1841**	Bbr_1841_Ird	cgacagcttccttgccatgc
**Bbr_1841**	Bbr_1841	gcgtgcgatgtccctgatg
**Bbr_1845/Bbr_1846**	*malFG/malR1*_Ird	cataacagcccctttgcc
**Bbr_1845/Bbr_1846**	*malFG/malR1*	catgactttcctcctccttgag
**Bbr_1847**	*malE*_Ird	ggaatgcctgagctgagccg
**Bbr_1847**	*malE*	cgaacctttctctttcatcgtcg
**Bbr_1891**	*gntR*_Ird	gatgagtgcgcgtgagaag
**Bbr_1891**	*gntR*	cacgctggcgaagattgtc
**Bbr_1894**	*PTS*_Ird	gatatgcgcgaggattgg
**Bbr_1894**	*PTS*	gatcgacatacagcatgccg
**Bbr_1901**	*nrdH* & *nrdI* & *nrdE_Ird*	gtctcgaacggcacacca
**Bbr_1901**	*nrdH* & *nrdI* & *nrdE*	tggacatccggtcaggcc
**Generation of DNA fragments for EMSA Fragmentations**		
**Bbr_0725**	*eno*_1_Ird	caaggaagtcgccgacaatc
**Bbr_0725**	*eno*_2	ggtgtgccgcgtgattgc
**Bbr_0725**	*eno*_3	ccaaaatttgatgtgagcgctc
**Bbr_0725**	*eno*_4	ggttgccacgagaatccag
**Bbr_0757**	*pyk*_1_Ird	cgctgagaaggcctgaaatc
**Bbr_0757**	*pyk*_2	gcgccgattgggttttgag
**Bbr_0757**	*pyk*_3_Ird	ccggtgtgttgtgagcgc
**Bbr_0757**	*pyk*_4_Ird	ggttgtcgtaatcctcggtg
**Bbr_0032/Bbr_0033**	*malR5/malE2*_1_Ird	cgccatgatgtctcctttcgc
**Bbr_0032/Bbr_0033**	*malR5/malE2*_2	caacgcgcacatcgtggtac
**Bbr_0032/Bbr_0033**	*malR5/malE2*_3_Ird	gtaccacgatgtgcgcgttg
**Bbr_0032/Bbr_0033**	*malR5/malE2*_4	cacaccgtcaaccgccgc
**Bbr_0032/Bbr_0033**	*malR5/malE2*_5	gcggcggttgacggtgtg
**Bbr_0032/Bbr_0033**	*malR5/malE2*_6	gctcatgtgatacgtgcctcc
**Bbr_0032/Bbr_0033**	*malR5/malE2*_7_Ird	gcagccggcatccgatcc
**Bbr_0112/Bbr_0113**	*malR6/agl3*_1_Ird	cgcttgaatgcttgcttttgc
**Bbr_0112/Bbr_0113**	*malR6/agl3*_2	gacaatcgaaacgcacacacc
**Bbr_0112/Bbr_0113**	*malR6/agl3*_3	ccaccgggcatgatacc
**Bbr_0112/Bbr_0113**	*malR6/agl3*_4	ccatgtcggcgaatttcctc
**Bbr_0112/Bbr_0113**	*malR6/agl3*_5	ccatgctattatgcaaacgatgtcag
**Bbr_0112/Bbr_0113**	*malR6/agl3*_6_Ird	ccggaacttctcgctatcatcatg
**Bbr_0122/Bbr_0123**	*malR3/apuB*_1_Ird	ggtagatgtctgccttgccc
**Bbr_0122/Bbr_0123**	*malR3/apuB*_2	gattacacatcgtggatggcgc
**Bbr_0122/Bbr_0123**	*malR3/apuB*_3_Ird	gcgccatccacgatgtgtaatc
**Bbr_0122/Bbr_0123**	*malR3/apuB*_4	gggaagtgttgcttggtgtgg
**Bbr_0122/Bbr_0123**	*malR3/apuB*_5	ccacaccaagcaacacttccc
**Bbr_0122/Bbr_0123**	*malR3/apuB*_6	cggcatgcagcacagttgac
**Bbr_0122/Bbr_0123**	*malR3/apuB*_7_Ird	gtcaactgtgctgcatgccg
**Bbr_0122/Bbr_0123**	*malR3/apuB*_8	ctgaccgtgcgatagggg
**Bbr_0122/Bbr_0123**	*malR3/apuB*_9	cccctatcgcacggtcag
**Bbr_0122/Bbr_0123**	*malR3/apuB*_10_Ird	gcaaggcgtttggcgagcg

Supplemental Table S3. Location of the AraQ/MalR1 TFBS in relation to the -10/-35 sites. The distance in base pairs [bp] is measured format the 3' -end of the TFBS to the 5' -end of the predicted -35 sequence.

Locus Tag Bbr_	Gene Name	Activator/ Repressor/ Unknown	Distance between TFBS and predicted -35 [bp]
0725	*eno*	Activator	16
0747	*carD*	Possible Repressor	-1 (overlaps with -35)
0757	*pyk*	Activator	15
0787	*pfl*	Activator	33
1002	*tkt*	Activator	6
1233	*gap*	Activator	35
1273	*ldh2*	Activator	65
1847	*malE*	Activator	92
1723	-	Activator	41
0037	*icfA*	Activator	34
0038	*ahpC*	Unknown	84
0117	*agl4*	Repressor	-9 (overlaps with -35)
0060	*glgP*	Repressor	-13 (overlaps with -10)

Supplemental Table S4. Effector molecules tested

Effector Molecule
Maltose
Maltotriose
Maltulose
Isomaltose
Lactose
Glucose
Galactose
Sucrose
Trehalose
Glucose 6-phosphate
Glucose 1-phosphate
Pyruvic acid
Sodium Acetate
Fructose 6-phosphate
Cellobiose
Palatinose
Turanose
DL-Glyceraldehyde 3-phosphate
Acetyl coenzyme A sodium salt
Phosphoenolpyruvate
Acetyl Phosphate
D-Sedoheptulose-7-phosphate
D-Ribose 5-phosphate disodium salt
D-(-)-3-Phosphoglyceric acid disodium salt
Butyrate
Lactate
Propioante
Acetate
Acetyl Aldehyde
1,2 Propanediol
D-erythrose-4-Phosphate
Oxaloacetic acid
Cyclic-AMP
Succinic acid
D-Ribulose 5-phosphate disodium salt

Supplemental Table S5.

Transcriptomic analysis of *B. breve* UCC2003-*araQ* (Up-regulation)
Global transcriptomic analysis of *B. breve* UCC2003-*araQ* mutant as compared with *B. breve* UCC2003 when grown on mMRS supplemented with ribose (up-regulated gene above a 2-fold threshold are indicated). The level of expression is shown as a fold-value of increase in expression, with a cut-off of a minimum >2-fold increase in expression.

Locus tag	Up	Gene name and/or predicted Function	P value
Bbr_0026	2.2	Permease protein of ABC transporter system for sugars	4.50E-01
Bbr_0027	2.2	Permease protein of ABC transporter system for sugars	4.53E-01
Bbr_0030	2.7	Conserved hypothetical protein	3.67E-01
Bbr_0060	2.6	*glgP1*, Glycogen phosphorylase	3.78E-01
Bbr_0110	6.5	*ilvC2*, Ketol-acid reductoisomerase/2-dehydropantoate 2-reductase	1.54E-01
Bbr_0116	2.5	*malQ*, 1 4-alpha-glucanotransferase	3.98E-01
Bbr_0117	2.8	*agl4*, Alpha-glucosidase	3.59E-01
Bbr_0118	4.4	*malE1*, Maltose/maltodextrin-binding protein	2.25E-01
Bbr_0119	4.4	*malF1,* Maltodextrin transport system permease protein	2.28E-01
Bbr_0120	4.5	*malG1*, Maltose transport system permease protein	2.21E-01
Bbr_0121	3.9	Conserved hypothetical membrane spanning protein	2.59E-01
Bbr_0122	3.7	*malR3*, Transcriptional regulator, LacI family	2.73E-01
Bbr_0123	4.3	*apuB,* Amylopullulanase	2.34E-01
Bbr_0164	4.3	*oppA1* Oligopeptide-binding protein	2.35E-01
Bbr_0165	4.6	*oppB1*, Oligopeptide transport system permease protein	2.18E-01
Bbr_0171	2.2	Sialidase A	4.60E-01
Bbr_0284	2.5	Sugar/Sodium symporter	4.04E-01
Bbr_0285	2.3	*lacZ2*, Beta-galactosidase	4.34E-01
Bbr_0538	2.2	*cysK*, cysteine synthase	4.63E-01
Bbr_1416	2.0	*rbsD*, D-Ribose pyranase	4.95E-01
Bbr_1429	2.1	*cbiO2*, Cobalt transport ATP-binding protein	4.70E-01
Bbr_1430	2.1	*cbiQ*, Cobalt transport protein	4.81E-01
Bbr_1530	2.8	Conserved hypothetical protein with CHAP and transglycosylase SLT	3.62E-01
Bbr_1644	2.3	Narrowly conserved hypothetical membrane spanning protein	4.35E-01
Bbr_1742	2.3	L-fucose permease	4.37E-01
Bbr_1743	2.3	Short chain dehydrogenase	4.38E-01
Bbr_1842	2.2	*aap6*, Amino acid permease	4.64E-01
Bbr_1845	2.4	Permease protein of ABC transporter system for sugars	4.24E-01
Bbr_1889	5.1	Cell surface protein with gram positive anchor domain	1.95E-01
Bbr_1890	6.2	ATP-binding protein of ABC transporter system for sugars	1.62E-01
Bbr_1891	7.6	Transcriptional regulator, GntR family	1.32E-01
Bbr_1892	8.6	PTS system, IIC component	1.16E-01

Supplemental Table S6.
Transcriptomic analysis of *B. breve* UCC2003-*araQ* (Down-regulation)

Global transcriptomic analysis of *B. breve* UCC2003-*araQ* mutant as compared with *B. breve* UCC2003 when grown on mMRS supplemented with ribose (down regulated gene above a 2-fold threshold are indicated). The level of expression is shown as a fold-value of increase in expression, with a cut-off of a minimum >2-fold increase in expression.

Locus tag	Down	Gene name and/or predicted Function	P value
Bbr_0104	4.0	*ilvC1*, Ketol-acid reductoisomerase/2-dehydropantoate 2-reductase	0.00E+00
Bbr_0113	3.6	Cell surface protein precursor with Cna protein B-type domain and	1.43E-08
Bbr_0114	2.3	Cell surface protein with Gram positive anchor and Cna protein B-type domain	0.00E+00
Bbr_0115	2.4	Sortase	1.48E-05
Bbr_0267	2.1	*glpF*, Glycerol uptake facilitator protein	7.11E-15
Bbr_0366	7.3	Narrowly conserved hypothetical secreted protein with Gram	0.00E+00
Bbr_0368	2.8	Conserved hypothetical protein	1.10E-08
Bbr_0441	14.1	Capsular polysaccharide biosynthesis protein	1.17E-11
Bbr_0442	11.7	Capsular polysaccharide biosynthesis protein	3.77E-15
Bbr_0443	6.3	Glycosyltransferase	1.66E-12
Bbr_0444	41.2	Membrane spanning polysaccharide biosynthesis protein	0.00E+00
Bbr_0445	8.7	Glycosyltransferase	0.00E+00
Bbr_0446	14.9	Acetyltransferase (cell wall biosynthesis)	2.33E-12
Bbr_0447	6.5	Conserved hypothetical protein	1.22E-15
Bbr_0448	4.3	Glycosyltransferase	4.05E-07
Bbr_0449	5.2	Hypothetical membrane spanning protein	5.71E-14
Bbr_0450	2.7	Membrane spanning protein involved in polysaccharide biosynthesis	1.44E-08
Bbr_0532	6.1	Transcriptional regulator, homologs of Bvg accessory factor	4.44E-16
Bbr_0533	3.3	Solute-binding protein of ABC transporter system for peptides	0.00E+00
Bbr_0534	2.7	Permease protein of ABC transporter system for peptides	0.00E+00
Bbr_0535	2.6	Permease protein of ABC transporter system for peptides	0.00E+00
Bbr_0536	3.0	ATP-binding protein of ABC transporter system for peptides	3.38E-12
Bbr_0602	4.0	Low specificity-threonine aldolase	1.19E-08
Bbr_0610	2.1	Conserved hypothetical membrane spanning protein	7.10E-08
Bbr_0611	2.4	Narrowly conserved hypothetical protein	3.37E-07
Bbr_0612	2.1	*crcB*, family protein	1.98E-07
Bbr_0674	2.4	Peptidase family M20A protein	7.44E-15
Bbr_0675	2.6	Permease protein of ABC transporter system	6.63E-11
Bbr_0889	2.3	Glutamine amidotransferase	1.48E-13
Bbr_0924	2.6	pntB NAD(P) transhydrogenase subunit beta	0.00E+00
Bbr_1078	2.1	Lantibiotic transport ATP-binding protein	3.23E-04
Bbr_1327	6.7	dTDP-rhamnosyl transferase	0.00E+00
Bbr_1328	4.2	Conserved hypothetical membrane spanning protein	0.00E+00
Bbr_1364	2.3	*groEL*, 60 kDa chaperonin	0.00E+00
Bbr_1474	3.0	Sua5/YciO/YrdC/YwlC family protein	0.00E+00
Bbr_1475	2.0	*livF*, Branched-chain amino acid transport ATP-binding protein	8.88E-16
Bbr_1476	2.1	*livG*, Branched-chain amino acid transport ATP-binding protein	2.47E-12

Bbr_1477	2.0	*livM*, Branched-chain amino acid transport system permease protein	3.12E-11
Bbr_1478	2.6	*livH* , Branched-chain amino acid transport system permease protein	5.95E-14
Bbr_1642	2.2	*rpsJ*, 30S ribosomal protein	1.58E-12
Bbr_1718	2.1	Hypothetical protein	5.55E-16
Bbr_1719	2.3	*fas*, Type I multifunctional fatty acid synthase	0.00E+00
Bbr_1720	2.1	*accD*, Acetyl-/propionyl-CoA carboxylase beta chain	2.22E-16
Bbr_1884	2.3	*galT2*, Galactose-1-phosphate uridylyltransferase	4.19E-06
Bbr_1886	2.1	Narrowly conserved hypothetical secreted protein	9.42E-12

Supplemental Table S7.
Transcriptomic analysis of *B. breve* UCC2003-*malR1* (Up-regulation)

Global transcriptomic analysis of *B. breve* UCC2003-malR1 mutant as compared with *B. breve* UCC2003 when grown on mMRS supplemented with ribose (up-regulated gene above a 1.2-fold threshold are indicated). The level of expression is shown as a fold-value of increase in expression, with a cut-off of a minimum >1.2-fold increase in expression.

Locus tag	Up	Gene name and/or predicted Function	P value
Bbr_0118	2	*malE1*, Maltose/maltodextrin-binding protein	3.61E-13
Bbr_0119	1.3	*malf1*, Maltodextrin transport system permease protein	2.46E-09
Bbr_0123	1.8	*apuB*, Amylopullulanase	6.34E-12
Bbr_0299	1.2	Holin	2.45E-07
Bbr_0391	1.2	*ilvB*, Acetolactate synthase large subunit	3.24E-07
Bbr_0558	1.3	Transcriptional regulator, LacI family	4.66E-12
Bbr_0607	1.4	*Tuf*, Protein Translation Elongation Factor Tu (EF-TU)	3.61E-03
Bbr_0725	1.3	*eno*, Enolase	2.93E-04
Bbr_0776	1.5	Xylulose-5-phosphate/Fructose-6-phosphate phosphoketolase	4.61E-06
Bbr_0787	1.2	*pfl*, Formate acetyltransferase	1.36E-01
Bbr_0847	1.3	*nagB2*, Glucosamine-6-phosphate isomerase	3.99E-10
Bbr_0848	1.3	Sugar kinase, ROK family	4.91E-06
Bbr_0921	1.3	*fadD2*, Long-chain-fatty-acid--CoA ligase	4.65E-09
Bbr_0969	1.3	*metE*, 5-methyltetrahydropteroyltriglutamate—homocysteine methyltransferase	6.99E-11
Bbr_0970	1.3	*metF*, Methylenetetrahydrofolate reductase	1.28E-12
Bbr_0973	1.3	*pyrB*, Aspartate carbamoyltransferase	5.89E-11
Bbr_1003	1.3	*tkt*, Transketolase	5.39E-04
Bbr_1079	1.3	Two-component response regulator	7.20E-11
Bbr_1233	1.3	Glyceraldehyde 3-phosphate dehydrogenase	4.01E-03
Bbr_1273	1.2	*ldh2*, L-lactate dehydrogenase	3.43E-04
Bbr_1367	1.3	*ung*, Uracil-DNA glycosylase	3.31E-11
Bbr_1505	1.2	*fucO*, Lactaldehyde reductase	2.05E-05
Bbr_1537	1.3	Hypothetical protein	2.53E-11
Bbr_1635	1.3	rpsC, 30S ribosomal protein	9.91E-03
Bbr_1649	1.3	*rplM*, 50S ribosomal protein L13	2.09E-02
Bbr_1719	1.2	*fas*, Type I multifunctional fatty acid synthase	6.48E-08
Bbr_1909	1.3	Conserved hypothetical protein	3.96E-08

Supplemental Table S8.

Transcriptomic analysis of *B. breve* UCC2003-*malR1* (Down-regulation)
Global transcriptomic analysis of *B. breve* UCC2003-malR1 mutant as compared with *B. breve* UCC2003 when grown on mMRS supplemented with ribose (down-regulated gene above a 1.2-fold threshold are indicated). The level of expression is shown as a fold-value of increase in expression, with a cut-off of a minimum >1.2-fold increase in expression.

Locus tag	Down	Gene name and/or predicted Function	P value
Bbr_0112	1.8	*malR6*, Transcriptional regulator, LacI family	7.65E-07
Bbr_0030	1.3	Conserved hypothetical proetin	1.63E-01
Bbr_0044	1.3	*pelF*, Glycosyl transferase (Polysacharide biosynthesis protein)	1.65E-08
Bbr_0045	1.2	Conserved hypothetical membrane spanning protein	4.09E-09
Bbr_0107	1.2	*cebF*, Cellobiose/cellotriose transport system permease protein	8.37E-11
Bbr_0468	1.2	Hypothetical protein	3.17E-10
Bbr_0535	1.3	Permease protein of ABC transporter system for peptides	5.61E-11
Bbr_1299	1.2	i*spA*, Lipoprotein signal peptidase	3.42E-07
Bbr_1482	1.3	*rpmE2*, LSU ribosomal protein L31P	2.97E-06
Bbr_1597	1.3	*rrf2*, family protein	3.53E-09
Bbr_1598	1.4	Pyridine nucleotide-disulphide oxidoreductase family protein	2.38E-14
Bbr_1658	1.2	Sugar-binding protein of ABC transporter system	2.32E-08
Bbr_1667	1.5	*rpmG*, LSU ribosomal protein L33P	3.67E-06
Bbr_1668	1.4	*Hsp10*, 10 kDa chaperonin GROES	1.24E-08
Bbr_1669	1.3	Narrowly conserved hypothetical membrane spanning protein	2.92E-10
Bbr_1731	1.3	*aspC*, Aspartate aminotransferase	3.75E-10
Bbr_1774	1.7	Transposase	3.19E-09
Bbr_1828	1.2	Transcriptional regulator, MarR family	4.07E-10
Bbr_1843	1.2	Narrowly conserved hypothetical membrane spanning protein	1.76E-07
Bbr_1844	1.3	Permease protein of ABC transporter system for sugars	2.67E-07
Bbr_1845	1.4	Permease protein of ABC transporter system for sugars	2.95E-08
Bbr_1854	1.3	*tdcB*, Threonine dehydratase	2.88E-10
Bbr_1873	1.3	Phospholipase/carboxylesterase	1.74E-11
Bbr_1890	1.2	ATP-binding protein of ABC transporter system for sugars	5.35E-04
Bbr_1905	1.3	Narrowly conserved hypothetical protein	7.05E-08
Bbr_1914	1.3	*pcnA*, tRNA nucleotidyl transferase	4.98E-09
Bbr_1918	1.2	*trxB2*, Thioredoxin reductase	3.41E-08
Bbr_1920	1.3	*parA*, Chromosome partitioning protein	7.06E-11
Bbr_1926	1.3	*rpmH*, LSU Ribosomal protein	6.81E-03

Supplemental Table S9.

Distribution of araQ and malR1 homologues among Bifidobacteriaceae members

Strain	araQ	*malR1*
Aeriscardovia aeriphila LMG 21773	+	0
Alloscardovia criceti DSM 17774 (*Metascardovia criceti* DSM 17774)	+	+
Alloscardovia macacae DSM 24762	+	+
Alloscardovia omnicolens F0580	+	+
Bifidobacterium actinocoloniiforme DSM 22766	+	0
Bifidobacterium adolescentis ATCC 15703	+	+
Bifidobacterium aesculapii DSM 26737 (DSM 26737T)	+	+
Bifidobacterium angulatum DSM 20098	+	+
Bifidobacterium animalis subsp. *lactis* AD011	+	+
Bifidobacterium animalis subsp. *animalis* LMG 10508	+	+
Bifidobacterium aquikefiri LMG 28769	+	+ (2x)
Bifidobacterium asteroides PRL2011	+	0
Bifidobacterium biavatii DSM 23969	+	0
Bifidobacterium bifidum NCIMB 41171	+	0
Bifidobacterium bohemicum DSM 22767	+	0
Bifidobacterium bombi DSM 19703	+	0
Bifidobacterium boum LMG 10736	+	+
Bifidobacterium breve UCC2003	+	+
Bifidobacterium callitrichos DSM 23973	+	+
Bifidobacterium catenulatum DSM 16992 = JCM 1194	+	+
Bifidobacterium choerinum LMG 10510	+	+
Bifidobacterium commune R-52791	+	0
Bifidobacterium coryneforme LMG 18911	+	+
Bifidobacterium crudilactis LMG 23609	+	+
Bifidobacterium cuniculi LMG 10738	+	+
Bifidobacterium dentium Bd1	+	+
Bifidobacterium eulemuris DSM 100216	+	+
Bifidobacterium gallicum DSM 20093	+	+
Bifidobacterium gallinarum LMG 11586	+	0
Bifidobacterium hapali DSM 100202	+	0
Bifidobacterium indicum LMG 11587 = DSM 20214	+	+
Bifidobacterium kashiwanohense JCM 15439 = DSM 21854	+	+
Bifidobacterium lemurum DSM 28807	+	+
Bifidobacterium longum subsp. *infantis* ATCC 15697 = JCM 1222	+	+
Bifidobacterium longum subsp. *longum* NCC2705	+	+
Bifidobacterium longum subsp. *suis* LMG 21814	+	+
Bifidobacterium magnum LMG 11591	+	+
Bifidobacterium merycicum LMG 11341	+	+
Bifidobacterium minimum LMG 11592	+	+
Bifidobacterium mongoliense DSM 21395	+	+
Bifidobacterium moukalabense DSM 27321	+	+
Bifidobacterium myosotis DSM 100196	+	+
Bifidobacterium pseudocatenulatum DSM 20438 = JCM 1200 = LMG 10505	+	+
Bifidobacterium pseudolongum PV8-2	+	+
Bifidobacterium pseudolongum subsp. *globosum* LMG 11569	+	+
Bifidobacterium pseudolongum subsp. *pseudolongum* LMG 11571	+	+
Bifidobacterium psychraerophilum LMG 21775	+	+
Bifidobacterium pullorum DSM 20433	+	0
Bifidobacterium reuteri DSM 23975	+	+
Bifidobacterium ruminantium LMG 21811	+	+
Bifidobacterium saeculare DSM 6531 = LMG 14934	+	0
Bifidobacterium saguini DSM 23967	+	+
Bifidobacterium scardovii JCM 12489 = DSM 13734	+	+
Bifidobacterium stellenboschense DSM 23968	+	+ (2x)
Bifidobacterium stercoris JCM 15918	+	+ (2x)
Bifidobacterium subtile LMG 11597	+	0
Bifidobacterium thermacidophilum subsp. *porcinum* LMG 21689	+	+
Bifidobacterium thermacidophilum subsp. *thermacidophilum* DSM 15837	+(2x)	+
Bifidobacterium thermophilum RBL67	+	+
Bifidobacterium thermophilum DSM 20212	+	+
Bifidobacterium tissieri DSM 100201	+	0
Bifidobacterium tsurumiense JCM 13495	+	+
Bombiscardovia coagulans DSM 22924	+	0
Gardnerella vaginalis 409-05	+	+
Gardnerella vaginalis ATCC 14019	0	+
Parascardovia denticolens DSM 10105 = JCM 12538	+	0
Pseudoscardovia radai DSM 24742	0	0
Pseudoscardovia suis DSM 24744	0	0
Scardovia inopinata JCM 12537 (*Scardovia inopinata* F0304)	0	0
Scardovia wiggsiae F0424	+	0
Total number of genomes with a gene	66	50
2x means 2 copies of a gene are present in a genome		

Supplemental Table S10. Oligonucleotide primers used in this study.

Purpose	Primer	Sequence
**Cloning of internal fragment of *malR1* in pORI19**	*malR1_IM_*	ttgctaaagcttgaaatcaggccgatcactgcg
	*malR1_IM_*	ttgctatctagacaatatcgcggtggccaagg
**Cloning of internal fragment of *araQ* in pORI19**	*araQ_IM_F*	atagctaagcttgattcgccggatgtgtctg
	araQ_IM_R	atagcttctagagtacagggcggacttatggc
**Confirmation of site specific homologous recombination**	*malR1_Con*	gccatcgccgacgaactc
	araQ_Con	gtgtgcaggccgccattg
	tetW_F	tcagctgtcgactgctcatgtacggtaag
	tetW_R	gcgacggtcgaccattaccttctgaaacat
**Cloning of *malR1* in pQE60**	*malR1_F*	ttgctaccatggatgaccacaagtatccaagatgtcgcc
	malR1_R	ttgctaggatccgcggatcttggcggtagagg
**Cloning of *araQ* in pNZ44**	*araQ_F*	atagcttctagaatggaggttcgggcagtatggtg
	araQ_R_hist	atagctaagcttttacatcaccatcaccatcaccat caccatcacgaacggcactcagcacagc

Purpose	Primer	Sequence
**Cloning of internal fragment of *malR1* in pORI19**	*malR1_IM_*	ttgctaaagcttgaaatcaggccgatcactgcg
	*malR1_IM_*	ttgctatctagacaatatcgcggtggccaagg
**Cloning of internal fragment of *araQ* in pORI19**	*araQ_IM_F*	atagctaagcttgattcgccggatgtgtctg
	araQ_IM_R	atagcttctagagtacagggcggacttatggc
**Confirmation of site specific homologous recombination**	*malR1_Con*	gccatcgccgacgaactc
	araQ_Con	gtgtgcaggccgccattg
	tetW_F	tcagctgtcgactgctcatgtacggtaag
	tetW_R	gcgacggtcgaccattaccttctgaaacat
**Cloning of *malR1* in pQE60**	*malR1_F*	ttgctaccatggatgaccacaagtatccaagatgtcgcc
	malR1_R	ttgctaggatccgcggatcttggcggtagagg
**Cloning of *araQ* in pNZ44**	*araQ_F*	atagcttctagaatggaggttcgggcagtatggtg
	araQ_R_hist	atagctaagcttttacatcaccatcaccatcaccat caccatcacgaacggcactcagcacagc

Supplemental Figures

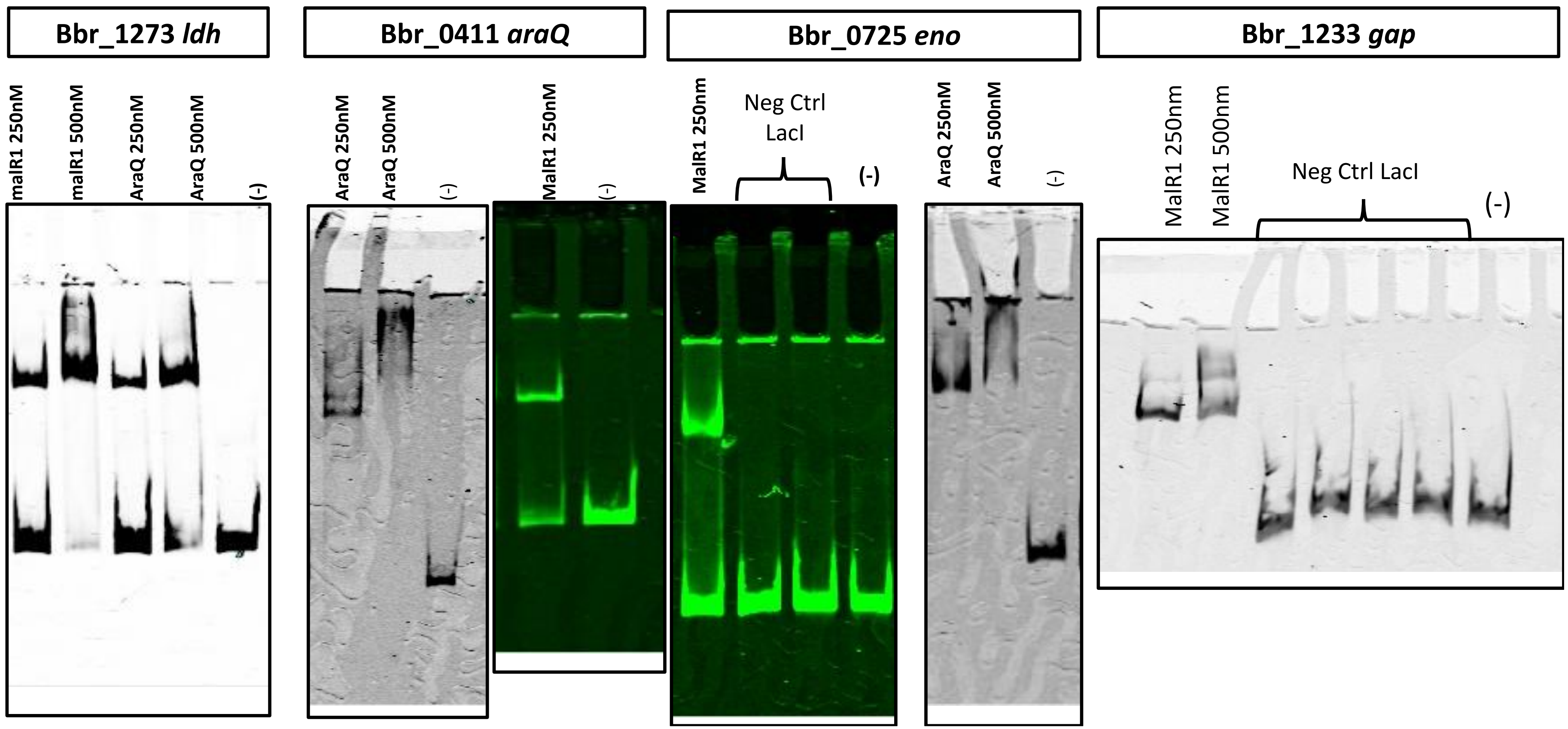

Figure S1. Example EMSA

All EMSA analysis carried out in the above figure were carried out with 0 nM (-), 250 nM or 500 nM of AraQ or MalR1 protein. These proteins were incubated with 0.5 nM Ird labelled DNA fragments encompassing the promoter region of the specified gene whose locus tag and gene name are indicated above each image in bold.

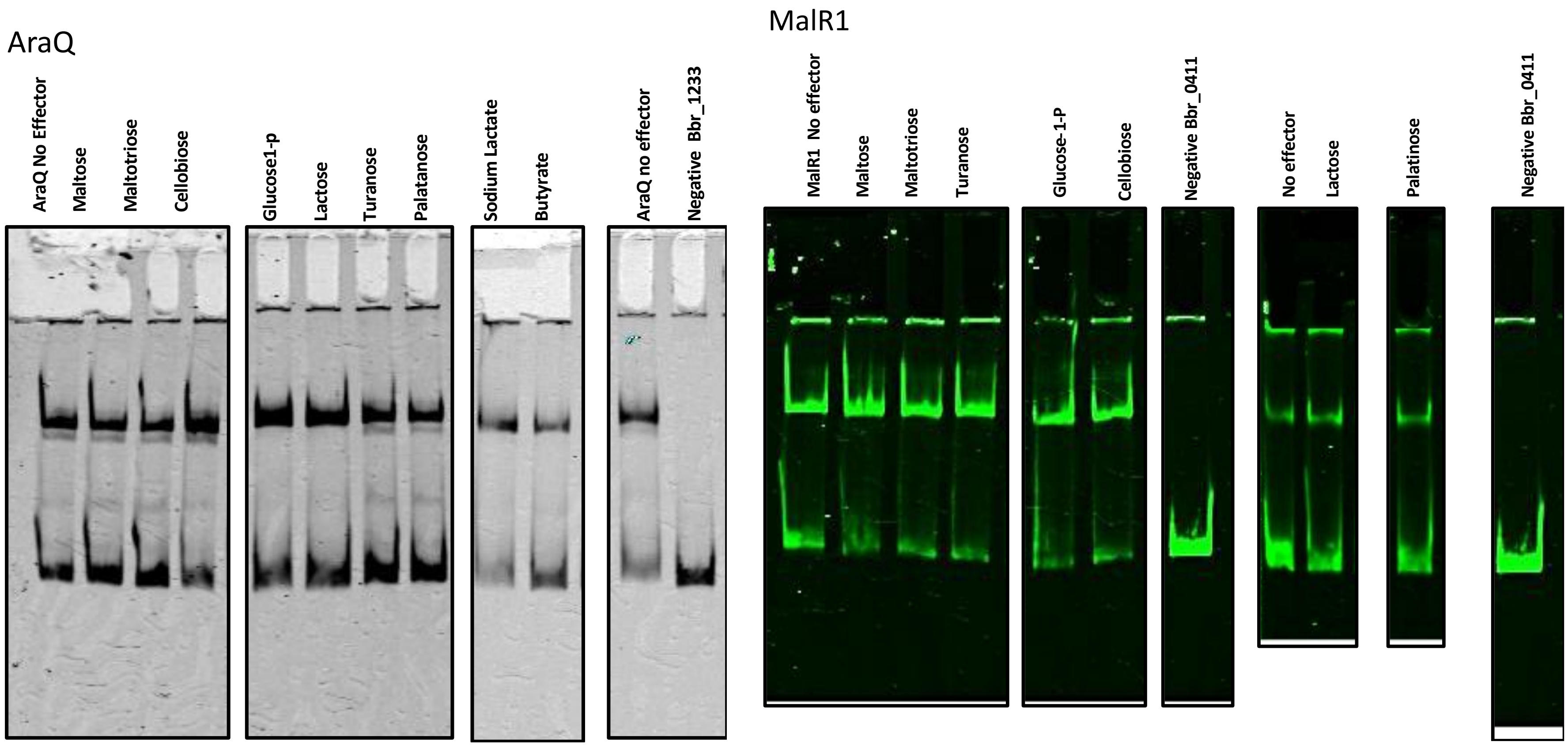

Figure S2. Example EMSA of AraQ and MalR1 Effector Assays

All EMSA analysis were carried out with 150 nM protein (AraQ or MalR1) or 0 nM protein (Neg.), 10mM Effector Listed above and with 0.5 nM Ird labelled DNA fragments encompassing the promoter region of the specified gene.

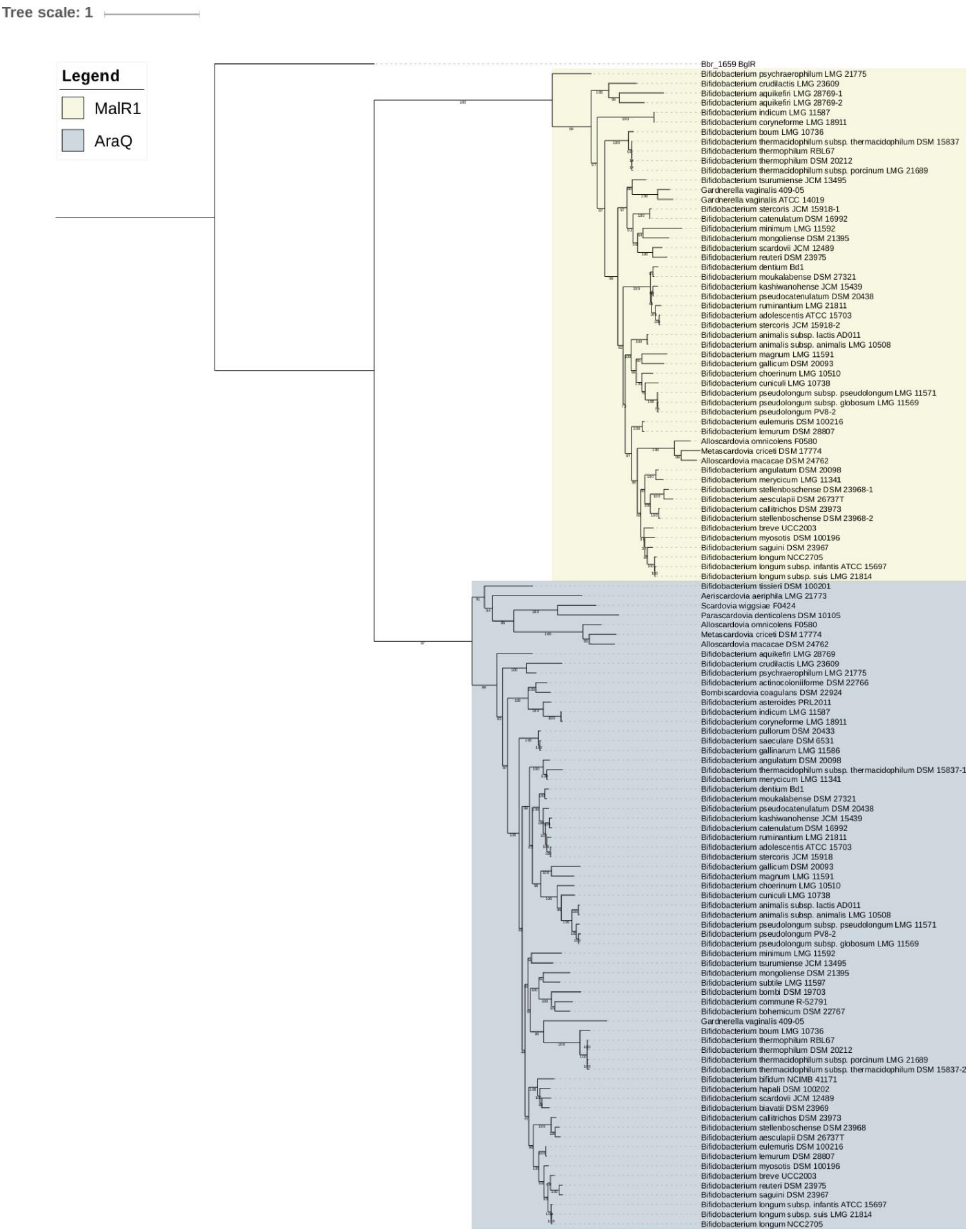

Supplemental Figure S3. Phylogenetic analysis of AraQ and MalR1

The analysis was carried out utilising an ultrafast bootstrap with 1000 replicates. BglR (Bbr_1659) a distant LacI family regulator from *B. breve* UCC2003, was utilised as an out group.

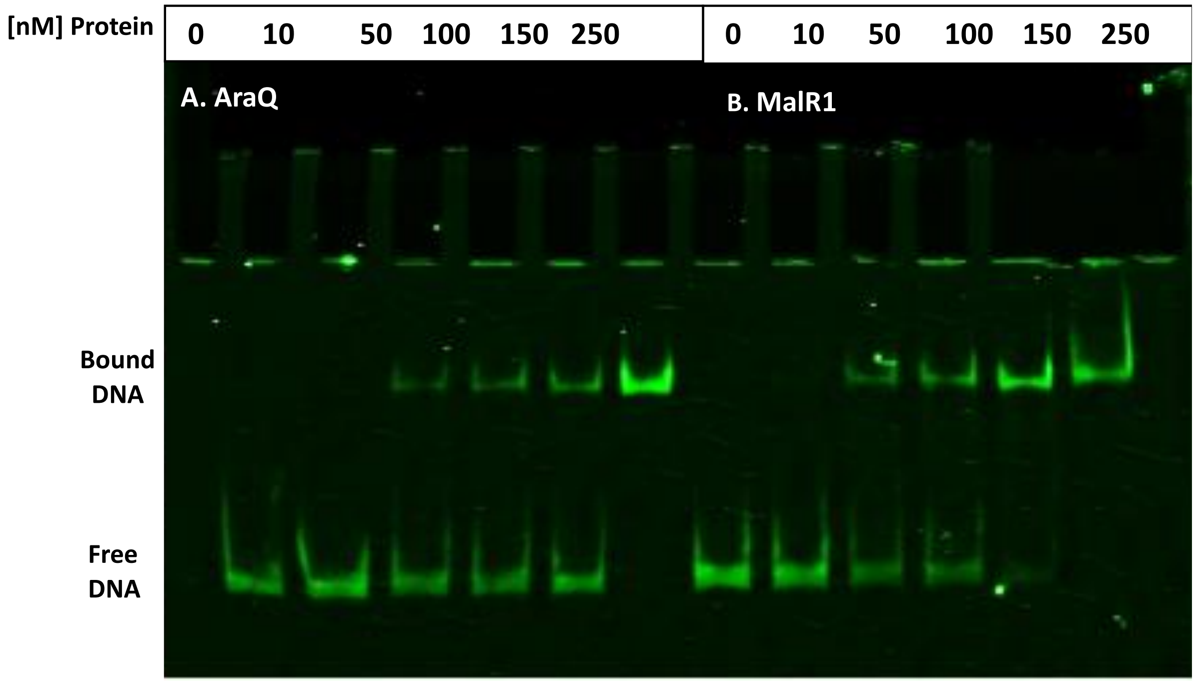

Supplemental Figure S4. Uncropped Image of gel in Figure 1.

EMSA carried out utilising Increasing concentrations (0, 10, 50, 100, 150, 250nM purified protein) of purified AraQ (A) and MalR1 (B) incubated with 0.5nM Ird labelled DNA fragment encompassing the Bbr_1233 promoter region.

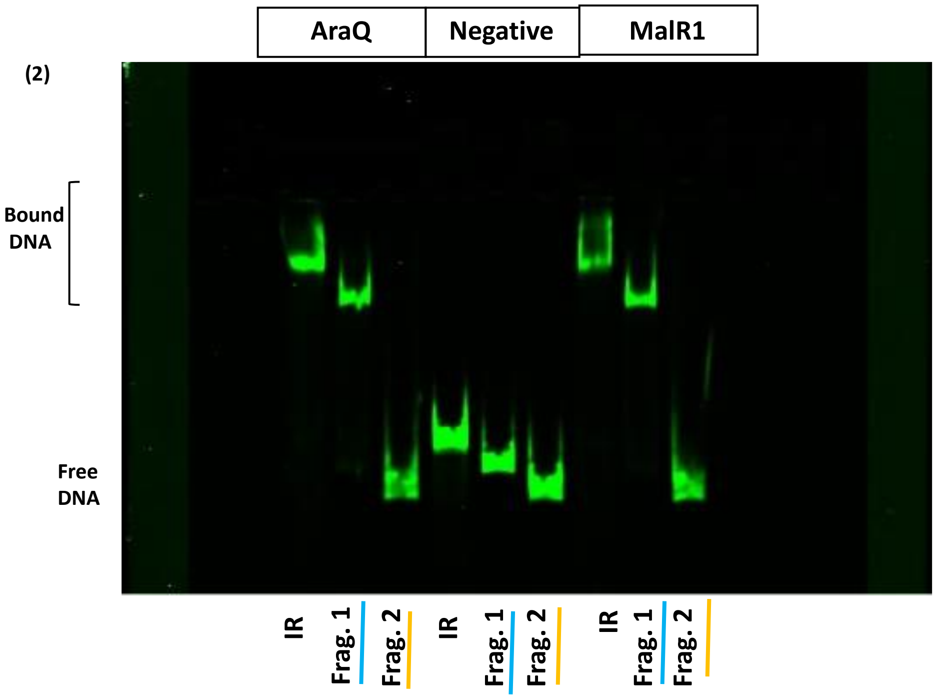

Supplemental Figure S5. Uncropped gel image of gel in Figure 3 Panel (2)

EMSA to investigate AraQ and MalR1 abilities to bind to Bbr_0757 promoter region fragments (IR, Frag 1 and Frag 2). All reactions contain 0.5 nM Ird labelled DNA and 150 nM of either AraQ or MalR1 protein, while negative reactions contain 0 nM protein.
